# Funding employment inclusion for Ontario youth with disabilities: a theoretical cost-benefit model

**DOI:** 10.3389/fsoc.2024.1281088

**Published:** 2024-04-10

**Authors:** Laura R. Bowman, Carolyn McDougall, René Doucet, Brendon Pooran, Ying Xu, Jeannette Campbell

**Affiliations:** ^1^Holland Bloorview Kids Rehabilitation Hospital, Toronto, ON, Canada; ^2^Occupational Science and Occupational Therapy, University of Toronto, Toronto, ON, Canada; ^3^Chronicle Analytics, Toronto, ON, Canada; ^4^Ontario Disability Employment Network, Whitby, ON, Canada

**Keywords:** employment participation, youth with disabilities, early intervention, cost-benefit modeling, societal-level benefit

## Abstract

Early engagement in employment-related activities is associated with greater lifetime labor force attachment, which correlates with positive health, social, and quality of life outcomes. People with disabilities often require vocational intervention to enter and remain in the workforce and reap the employment-related health and social benefits. Their labor force attachment brings about the added societal-level benefits of increased tax contributions and reduced social assistance funding. Reason and evidence both support the need for early intervention to facilitate young people with disabilities’ workforce entry. Based on available evidence and best practices, and in conjunction with expert input, a cost–benefit model was constructed to provide support for public investment in early employment intervention by demonstrating the societal-level benefits that could be projected. Results indicate the potential benefits for investment in early, targeted employment intervention at a societal level. Two personas were crafted to demonstrate the lifetime societal-level impact of investment in intervention for an individual with disabilities. The results provide relevant arguments for advocates, policy makers, program directors, and people entering adulthood with disabilities to understand the benefits of investing in interventions with the goal of long-term public savings.

## 1 Introduction

Persons with disabilities represent the largest Canadian population who are unemployed and searching for employment. According to Statistics Canada, this group has a national employment rate of 59%, as compared with 80% of adults without disabilities (Morris et al., 2018). This rate is even lower (26.1% employment) when specifically considering those with intellectual and developmental disabilities (Berrigan et al., 2023). Yet, workforce participation of people with disabilities, a group representing diverse characteristics, skills, and support needs, is beneficial at multiple levels. For the people with disabilities themselves, employment participation is linked with greater lifetime financial stability and reduction of poverty (Brucker et al., 2015), social connection, and mental stimulation (Jacob et al., 2015). People with disabilities who have employment have also demonstrated lower use of healthcare services (Thomas and Ellis, 2013), better overall quality of life, and lower use of public social assistance funding than those without employment (Hall et al., 2013; Tompa et al., 2021).

Employers and organizations also benefit from hiring and retaining employees with disabilities who bring with them unique skills, innovative ideas, and the ability to connect with diverse market segments (Lindsay et al., 2018; Scott et al., 2019). Organizations benefit from overall lower employee turnover, increased consumer diversity and profits related to visibility of an inclusive workforce, and creativity and innovation on the part of a population who must regularly problem-solve and seek creative solutions (Lindsay et al., 2018; Ontario Disability Employment Network, n.d.). For employers facing actual or potential domestic labor market shortages, this underemployed population represents a viable and skilled group of potential employees. At a societal level, increased employment participation and greater representation of people with disabilities in the workforce has been connected to decreased social assistance expenditures (Beyer, 2017; Anderson et al., 2019; Scott et al., 2019), lower expenditure on community and social programming (Jacob et al., 2015), increased taxes contributed from employed workers (Anderson et al., 2019; Schochet, 2021), and increased ability to fill specialized labor force roles (Beyer, 2017).

The clear, permeating benefit of greater employment engagement for people with disabilities begs the question of how to increase their inclusion in the Canadian workforce. This requires multi-level considerations to create workers, workplaces, and workforce structures that are inclusive and appropriate. For the people with disabilities themselves, some of the strongest evidence-based best practices for labor force attachment include a ‘start-early’ approach (initiating workforce participation interventions during high school) (Liu et al., 2014; Wehman et al., 2015; Krahn and Chow, 2016; Kohler et al., 2017; Bowman et al., 2023). The benefits of the start-early approach are the initiation of skill-building during a developmentally relevant period, and increasing the lifetime workforce attachment and resulting benefits seen by individuals, employers, and society (as outlined above). Such interventions require strong, evidence-based disability employment support services, which are defined as follows, “[w]ith an emphasis on matching an individual with an appropriate employer and work environment, it involves individualized, rapid placement and ongoing support, training, and assessment that take into account the person’s vocational and personal needs” (American Psychological Association, 2023).

Through this article, we argue for increased availability of early employment support services for youth with disabilities to promote their entry into the workforce. To improve the quality of and equitable access to such programming, public investment in said programming is necessary. We will demonstrate a potential return on investment of early public funding for employment support services for youth with disabilities by modeling a theoretically-based cost benefit analysis. The model will consider person-based scenarios representing early investment in employment participation vs. inaction (maintaining the current state or status quo). Through this work, we intend to demonstrate the potential economic benefit of changing a behavior. We are not assuming that the behavior can be easily changed, rather that the potential return justifies further work in understanding if and how this behavioral change could be effected. Our hope is for the results of this paper to inform programs, funding streams, and the collection of reliable evidence to support ongoing funding when advocating for and with people with disabilities seeking employment, employers seeking to fill positions, and society seeking to be more inclusive and use resources effectively.

## 2 Methods

Due to a dearth of tangible, publicly available data on universal costs and benefits related to lifetime employment participation for people with disabilities in Canada, we undertook an integrated knowledge translation (iKT) project to construct a realistic model approximating the costs and benefits of early public investment in employment participation. The goal of the iKT process was creating a *starting point* from which contributors could explore, debate, and facilitate change in employment support services via the co-construction of a realistic and meaningful theoretical model. We worked with experts in the field of employment participation for people with disabilities, including youth with disabilities and their family members, service providers (occupational therapists, therapeutic recreation specialists, life skills coaches, vocational coaches), program administrators, funders, educators, advocates and advocacy organizations, policy makers, policy analysts, and researchers.

Small group meetings and a knowledge mobilization event were held to explore the current state of practices, policies, and lived experiences contributing to the Ontarian employment participation context. As outlined in the introduction of the paper, this population has among the highest levels of unemployment in Ontario. Youth with disabilities who are seeking employment while enrolled in high school or post-secondary education face unique barriers. Namely, they typically lack eligibility for and access to the same supported employment services available to job seekers with disabilities who are no longer enrolled in educational programming. Examples of these supported employment services include job development, job coaching and/or personal (attendant) care.

Based upon the discussions and inputs from our network of collaborators, the authors of this paper constructed a theoretical cost–benefit model that accounted for the public costs (paid out by government) and benefits (public funds recovered by government) for people with disabilities over their lifetime. The cost–benefit model calculates government-level outputs (refund or balance owing) for *a single individual at any income level*. All inputs, calculations, and results therefore refer to the lifetime public refunds/balances owing per person.

### 2.1 Model construction

In keeping with the Treasury Board of Canada’s (2007) Cost Benefit Analysis Guide, we established a baseline scenario and compared intervention-specific scenarios and outcomes with the baseline scenario to calculate relative net benefits. The scenarios across which our inputs were considered were:

1. *Baseline*: Status quo, no employment support intervention while in school but typical disability employment supports accessed after school completion, stable employment path projected into the future;
2. *Moderate*: An employment support intervention while in school that results in ‘as-expected’ post-program employment outcomes for the person, with a stable employment path projected into the future (during which timeframe individual also access typical disability employment supports); and
3. *Strong*: An employment support intervention accessed while in school that results in better than expected post-program employment outcomes for the person, with a stable employment path projected into the future (during which timeframe individual also access typical disability employment supports).

We note here that across our scenarios, being “in school” includes high school or postsecondary education, during which time youth are ineligible for public employment support funding. We calculate the net present value (NPV) of the cash flows of each scenario to obtain the relative net benefits and then divide the result by the cost of the intervention to produce a return. The calculations represent calculable lifetime costs and returns to government per person who would be eligible for employment support services. The model’s variables were designed based upon information drawn from Ontarian and Canadian public data (i.e., tax, social assistance). Input formulas were designed to account for variability in the types of employments, social assistance, and personal factors (e.g., support needs, family connections, accommodations, leaves) that influence individual employment experiences over the lifetime. The widely used discounted cash flow (DCF) analysis was used, whereby costs and benefits for a given scenario are projected out into the future and then discounted back to present day dollars. The modeling formula for our inflation-adjusted net present value calculation is in Table 1. An in-depth description of the formulas and inputs used to construct the model are available in Supplementary Appendix A for the sake of both transparency and promotion of further work of this nature.

**Table 1:** Modeling formula for inflation-adjusted net present value calculation.

Overall formula for return	$Return=\frac{NPV\;CF\;ProgramScenario-NPV\;CF\;BaselineScenario}{PV\;ProgramCost}$
NVP calculation	$NPV=\overset{N}{\underset{n=0}{\sum }}\frac{\left(IAIT_{n}-IATC_{n}\right)-\left(ODSPES_{n}+ODSPIS_{n}\right)}{{\left(1+r_{n}\right)}^{n}}$
NPV	Net Present Value
IAIT_n_	Inflation Adjusted Income Tax for year *n* which is used to calculate the annual tax revenue (Tax Revenue = *IAIT_n_* – IATC_n_)
IATC_n_	Inflation Adjusted Tax Credits for year *n* which is subtracted from IAIT to calculate the annual tax revenue (Tax Revenue = IAIT_n_ – *IATC_n_*)
ODSPES_n_	Ontario Disability Support Program (ODSP) Employment Supports for year *n* which is used to calculate the annual social assistance costs (Social Assistance = *ODSPES_n_* + ODSPIS_n_)
ODSPIS_n_	ODSP Income Supports for year *n* which is used to calculate the annual social assistance costs (Social Assistance = ODSPES_n_ + *ODSPIS_n_*)
*r*_n_	Discount rate
N	Number of years working in a lifetime in a given scenario

We remind readers that in our model we look strictly at the costs and benefits accruing to government per person, not to the hypothetical individuals accessing programs themselves or to their potential employers. In theory, this will yield a conservative return, as the model accounts for all of the program costs but does not account for all the benefits – both direct and indirect – experienced by individuals and businesses. A summary of the model’s variables and data sources for application is available in Table 2.

**Table 2:** Summary of model construction variables and application data sources.

Model construction		Model application
Global Inputs	Inflation rate Discount rate	Static across scenarios/personas, overarching variables, publicly available data
Local Context	Wages (minimum, industry standard) Employment Insurance information Federal/provincial income tax rates Federal/provincial income tax brackets Disability-related tax credit Program costs Social assistance costs (e.g. ODSP-related)	Context specific, stable across scenarios but may vary between personas, mix of publicly available data and expert insight
Persona: Tax Strategy	Pension-based variables Disability-related employment expenses Childcare expenses	Persona-specific, may change across scenarios, mix of publicly available data and expert insight
Persona: Earnings	Number of years working Hours/week Working weeks/year Wage or salary Alternate community supports Earnings-based pension exemptions	Scenario-specific, mix of publicly available data and expert insight

Before considering the model application, we will highlight that the social assistance calculation engine for our model is regionally specific. Our social assistance costs are in the form of ODSP Employment Supports and Income Supports, obtained from the relevant Ontario government websites (Ontario Ministry of Children, Community and Social Services, n.d.). We assume in our scenarios that *all available* ODSP employment supports (ODSP ES) and income supports (ODSP IS) would be claimed if an employer and/or individual were eligible to receive them. We thus built a social assistance engine that calculates both ODSP amounts paid out by the province, which represent a cost to government.

ODSP ES^1^ are the amounts paid by the government *to a service provider* that has placed a person into employment and/or helped them retain the employment, according to a milestone payment schedule. We assume conservatively that personas in the intervention scenarios outlined later will make full use of ODSP payments, even though they may have already transitioned to employment without the need of service providers, due to having been involved in the intervention. Detailed calculations are available in Supplementary Appendix A. ODSP IS are the amounts paid by the government *to the individual* to support basic living expenses. For modeling purposes, we conservatively assumed that each individual, regardless of the persona used, was single, had no dependents, and received food and shelter from a parent or other supportive person. The implication of this assumption is that the person is receiving the lowest possible amount of income supports, which is the “Board and Lodging” amount. In practice, we support independence, autonomy, and active social engagement for people with disabilities, but the assumption of living with caregivers was made to support the most simplified and conservative presentation of our model in terms of tax calculation. The detailed calculation for Income Supports is included in Supplementary Appendix A.

### 2.2 Model application

The model was designed so that it can be applied to any given local context to evaluate employment support interventions, with seven required variables and two optional variables that must be set (Table 3). The remaining variables were set as a function of the rules and standards associated with both the tax and social assistance regimes in Ontario, or by historical wage rates (see Supplementary Appendix A for details).

**Table 3:** Required and optional variables.

Required earnings variables	Static	Lifetime working years
		Wage in first working year
	For each working year	Weeks per year
		Hours per week
		Wage in respective year (reflects progression)
Optional variables		Deductible retirement contribution (RRSP^*^)
		Disability-related tax credits claimed (DTC^†^)
Required overarching variables		Inflation rate
		Discount rate

Required variables highlighted in light grey, optional variables highlighted in dark grey; *RRSP, Canadian Federal Registered Retirement Savings Plan; ^†^DTC, Canadian Federal Disability Tax Credit.

Once the theoretical cost–benefit model was constructed, our network of collaborators co-constructed realistic personas to whom the model was applied. Personas are fictional examples of typical target users that remain realistic in terms of how individuals will interact with the funding model (Harley, 2015). Two unique personas (Table 4) were created and refined by the group to drive decisions based on the needs of actual people rather than generic or undefined criteria. The personas constructed for this project were designed to reflect some of the characteristics we see through the intervention programs that we offer, and specifically chosen to reflect how the model can be applied to differing pathways to employment for people with disabilities. The intervention programs were chosen as examples of evidence-informed and evidence-based programs that demonstrate results related to start-early employment participation for people with disabilities.

**Table 4:** Persona descriptions at time of initiating intervention.

	High school persona	University persona
Age	20	16
Education	Entering final year of high school; non-credit course stream	Entering grade 11; Planning to go to university, interested in studying business
Diagnostic profile	Intellectual disability + Autism	Cerebral palsy affecting right arm/ leg; Mild learning disability
Public funding	ODSP Income Support	Will access ODSP Income Support when eligible (age 17.5)
Current employment participation	Chores at home; School-based co-operative education.	Chores at home; Community service hours (volunteering) through school events and local faith organization
Challenges and concerns	*Shyness*: Speaks inaudibly to unfamiliar people. Sometimes does not respond to greetings and never initiates. *Learning*: Learns best with repetition. Likes rules. ~Grade 2-level literacy/ numeracy. Difficulty estimating and tracking time. *Questions*: Family is hopeful they can work but unsure how or what kind of job.	*Physical*: Difficulty standing/walking for long periods. Unable to lift/carry heavy items. *Learning*: School accommodations re: organization skills, extra time for exams/assignments. *Questions*: Has questions about what type of job can do and how to tell employer about disability. Family is encouraging experience but has questions about inclusive employers.
Disability employment support intervention accessed while in school	**Project SEARCH** 10+ months immersed at business site while formally enrolled in high school (Wehman et al., 2020) *Staff support*: 1 teacher, 2 skills trainers (job coaches) shared by 10 students *Work experience*: 700+ hours total accrued over 3 placements/training rotations/“internships” (each 10 weeks long). Focus on determining job preferences and building skills *Life skills training*: daily classroom curriculum (1 h) + on the job skill-building *Other supports*: Coaching for self-discovery; employment planning *Pre-program preparation:* Transit training *Post-program:* Employment support (ODSP employment support program) **Coaching/Training Supports** *Project SEARCH:* Support during on-the-job training as needed. *Post-Program*: Will access job coaching again when starting new job(s)/as needed	**Employment Participation Pathway** Programs occurring over 2–3 years during high school (Bowman et al., 2023) *Staff support (as needed):* Life skills coach, occupational therapist, youth/family facilitators *YEAR 1: Youth@Work program* – Participated summer after grade 10. Supported volunteer work experience at hospital, includes 50 h in 2 roles; job coaching; self-discovery activities; workplace life skills workshops *YEAR 2: Employment Action Coaching* – Participated during grade 11. Included job search workshops; coaching to set personal employment goals and develop action plan *YEAR 2 and 3: Ready to Work program* – Participated summers after grades 11 and 12. Support to apply to summer job, disclose disability, request accommodations and get started. **Coaching/Training Supports** *When in Pathway:* Occupational therapist/coach available to support job development and start-up phases *Post-Pathway:* Transition to ODSP employment support agency if needed
Employment-related outcomes	Ontario Safe Food Handler Certification Job in food service industry: Entry at average industry wage (above minimum wage) ($17.35/h) Possible move to salaried position ($32,300.00/year)	Job at university book store, part-time, 3 years at minimum wage ($15.50/h) Summer job at bank, 1 year + new graduate program ($23.00/h) Bachelor of Business Administration degree Bank Relationship Manager, salaried position ($65,000.00/year)

*Personas constructed based on amalgam of clients taking part in listed interventions; Persona information reflects their experiences/profiles at the initial point of intervention unless otherwise specified.

The first persona represents perspectives from those who do not choose to attend or do not have access to post-secondary credentialing (“high school persona”). In the case of the second persona, the individual will access higher education and employment participation during school breaks before graduating (“university persona”). The personas and their outlined scenarios provide a realistic template and common focus for the construction and evaluation of the cost–benefit model and situate potential sensitivity analyses by grounding changes in the nine parameters listed in Table 3, as confirmed by our expert collaborators.

As outlined at the top of Table 1, baseline and program (intervention) scenarios must be established to calculate the return. Baseline scenarios for each persona reflect both the current and projected status quo over the lifetime of the model. The baseline scenarios therefore take the information available in Table 4, excluding the bottom two sections of ‘Early Employment Intervention’ and ‘Employment-related Outcomes’.

The intervention for the high school persona – Project SEARCH (Wehman et al., 2020; Project SEARCH, 2021) – was chosen as an evidence-based example that is currently being practiced in Ontario, Canada. For the university persona, there are currently few evidence-backed programs available that are being implemented in Ontario, and so we chose a locally-relevant, clinically-supported, evidence informed intervention – the Employment Participation Pathway (Bowman et al., 2023).

We constructed the baseline scenarios for both the high school and university personas to assume sustained employment over the lifetime of the person in question (Treasury Board of Canada, 2007). We estimate this to be a conservative assumption, given the research indicating that failure to attach to the labor market early lowers average lifetime earnings and reduces the likelihood of *ever* reaching a state of stable employment (Krahn and Chow, 2016). The research-based implication is that stable lifetime employment is not the common reality, but will provide a more conservative and stable estimate that would provide an even greater net benefit to government. Earnings variables (Table 3) were chosen as they related to the persona in question, and were meant to reflect the expected status quo over the lifetime of the model so as to be reasonable and conservative estimates.

In order to project earnings into future years, we use an inflation rate of 2.2%, representing the Bank of Canada’s (Bank of Canada, n.d.) target of 2% with a slight upwards adjustment to reflect the fact that the historic 60-year average exceeded the 2% target (World Data, n.d.). We applied the Social Discount Rate of 3% as recommended by the Canadian Cost–Benefit Analysis Guide for social projects (Treasury Board of Canada, 2007).

## 3 Results

The cost–benefit model was applied to each of the two personas described in Table 4, using the calculations presented in Table 1. Tables 5, 6 present summaries of the scenarios and relevant public funds cash flow as gross numbers and as differences from the baseline scenarios in the high school and university personas, respectively. Details of the modeling are available in Supplementary Appendix B.

**Table 5:** High school persona’s main results summary.

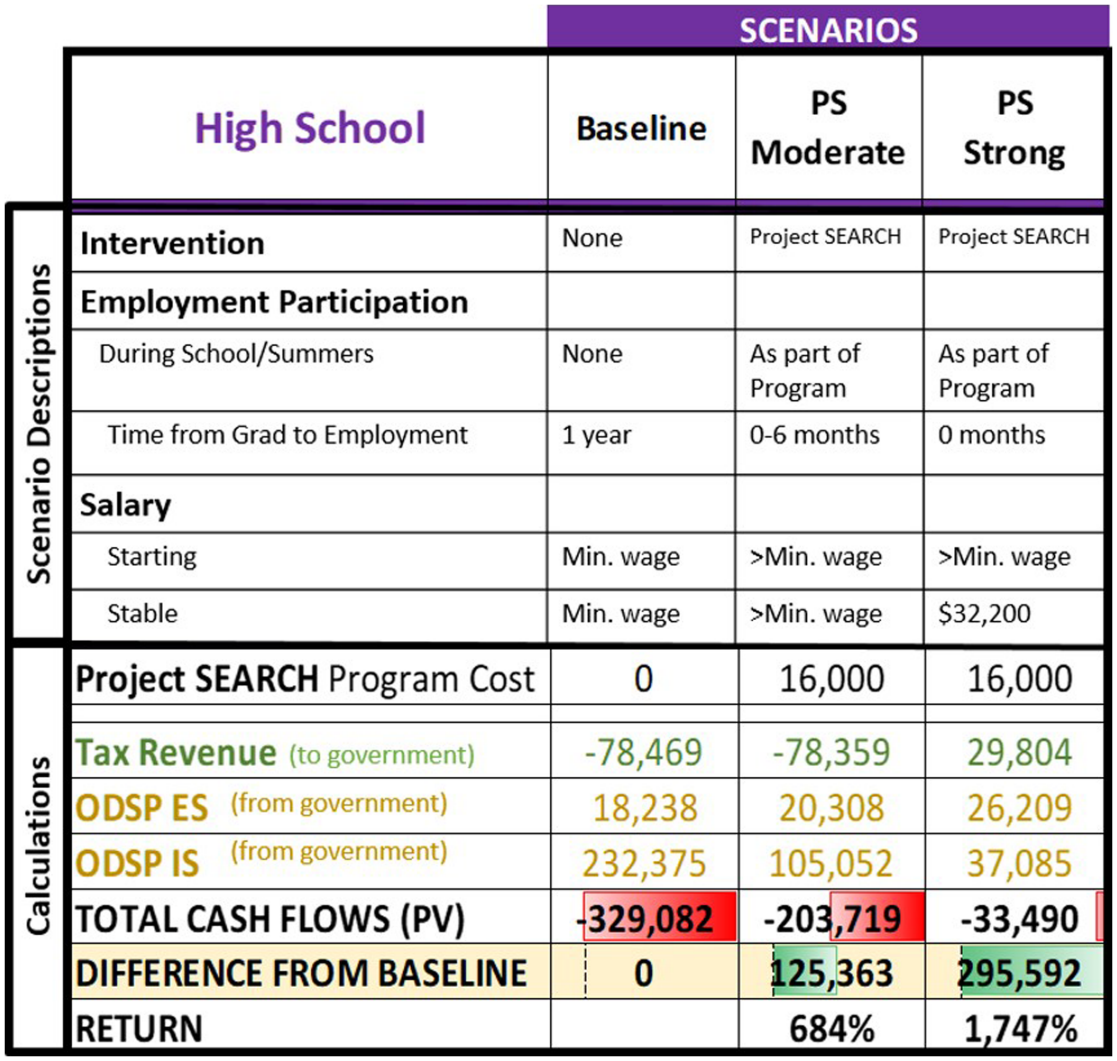

**Table 6:** University persona’s main results summary.

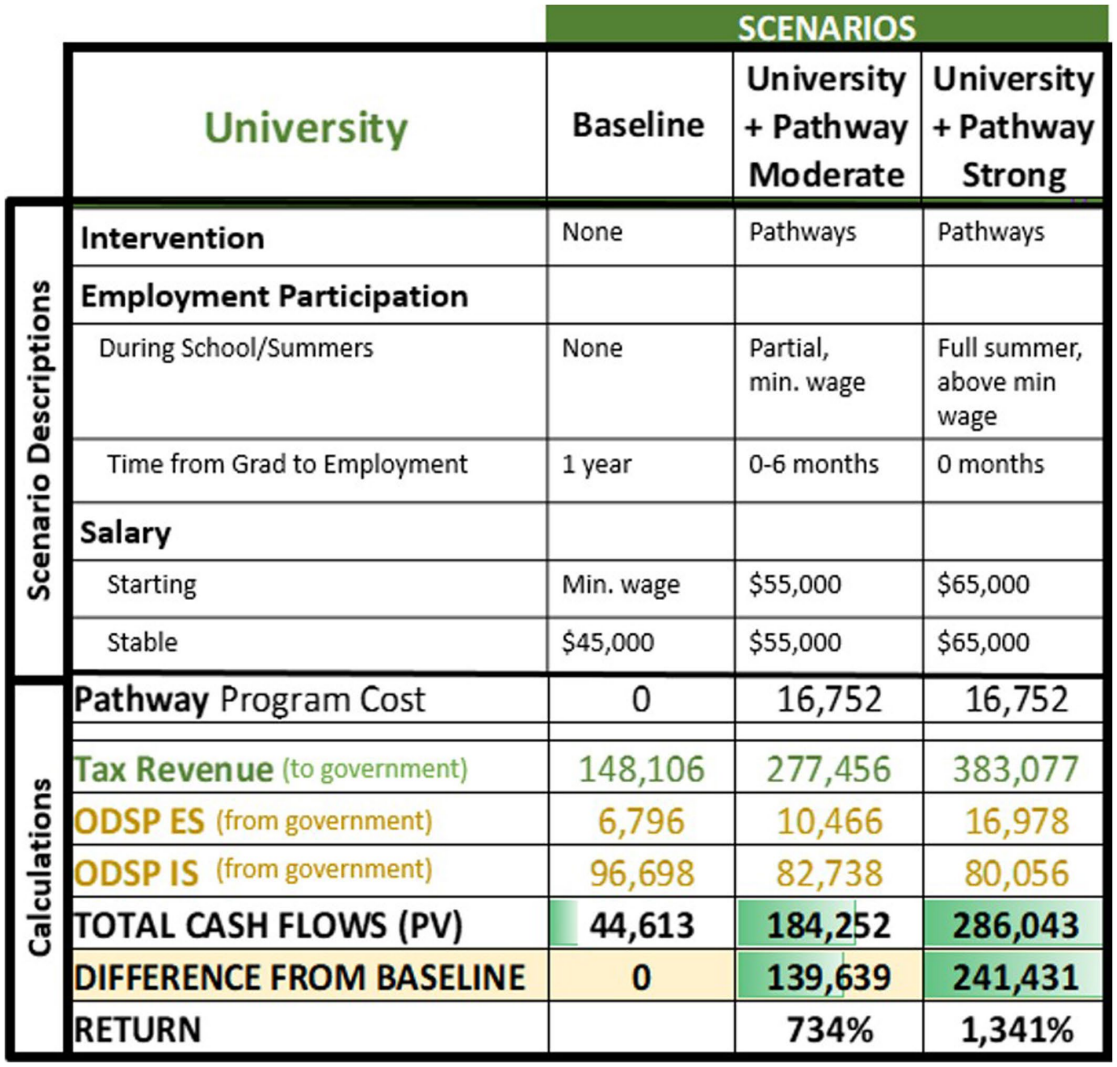

The high school persona was considered under the three listed scenarios. The persona was modeled under all scenarios to have the person work continuously until age 65 from the time of obtaining employment. The baseline scenario represents no intervention while in school (did not participate in Project SEARCH program), and that the person accessed employment supports otherwise available to a person with their disability and employability profile after completing high school. In this scenario, the person found a part-time job at minimum wage ($15.50/h) approximately 1 year after leaving high school (age 22). This timing was chosen based on a conservative application of the authors’ clinical experiences, Canadian data regarding earnings by age group and degree (Statistics Canada, 2022), and the previously indicated research indicating reduced lifetime labour force attachment without early work experiences (Krahn and Chow, 2016). The moderate and strong outcomes were derived from the literature on reasonable outcomes following participation in the Project SEARCH program (Wehman et al., 2020; Riehle and Team, 2022; Stakeholder Input). The moderate outcome scenario represents a person who found part-time work slightly above minimum wage ($17.35/h, 2021 average wage for the food services industry in Ontario) (Statistics Canada, 2022). The job was acquired after approximately 6 months of searching. The strong outcome scenario saw a greater number of hours with job attachment immediately post-program at the same above-minimum-wage rate, and a switch to a salaried position ($32,300) after 6 years.

Participation in the Project SEARCH program required an initial public investment of $16,000 per person, which was calculated based on the total cost of running the program,^2^ divided by 10 participants (a typical group size in Ontario). The negative amount of taxes paid (Table 5) reflects government issued refunds due to tax credits offsetting the taxes owed by the individual, resulting in a net flow from government to the person. The ODSP social assistance costs also represented payments *from* government *to* individuals or employers, and relate to overall reduced government spending. As labor force attachment rose, the ODSP ES (payment to employers) rose minimally, which supported ongoing employment and the creation of a scenario with reduced ODSP IS lifetime payments from government and increased lifetime income tax payments to government. Despite the overall negative cash flow across the intervention scenarios, there was an improvement compared to baseline. This outcome demonstrates overall government savings per person as compared to the baseline (current expected state).

The university persona was also considered under the three scenarios. The baseline scenario represents no intervention while in school (did not participate in Pathways programs), with the person accessing employment supports otherwise available to an individual with their disability and employability profile after they completed their high school and post-secondary education. In this scenario, the persona did not participate in paid employment during high school or university, and started searching for a job after graduation from university. They found a job after approximately 1 year of searching, starting at minimum wage and moving to a salaried position ($45,000) in the finance field after 5 years. In the moderate outcome scenario, the person participated in Pathway programs (Bowman et al., 2023; Stakeholder Input) during high school and worked during part of the summers for minimum wage during high school and university. They obtained a salaried position ($55,000) after university graduation in their preferred field of finance. In the strong outcome scenario, the person participated in Pathway programs during high school and worked during the full summer for above minimum wage ($23.00/h) during high school and university. They obtained a salaried position ($65,000) after graduation in their preferred field of finance with a higher starting rate than the moderate outcome scenario given their more extensive work experience. In both the moderate and strong outcome scenarios, the persona accessed typical disability employment supports after school completion upon entering their full-time jobs.

Participation in the Pathways program required an investment of $16,752 over two years. This was calculated based on hospital records to represent the upper range of program costs that vary annually by number of program hours per year, number of youth and staff per program, and average staff salary, and then multiplied by the 2 years of Pathway program participation used by our University persona. Lifetime tax revenues to government were greater for the two intervention scenarios, which each assume higher wages and more hours worked over more years than the baseline scenario. Slightly greater ODSP ES was paid out for the two intervention scenarios, given the greater number of hours worked initially. Slightly less lifetime ODSP IS was paid out due to higher overall employment earnings because disability income support payments are reduced with higher employment earnings. The total cash flow that included cost of intervention and supports paid out minus the taxes recouped by the government represented a net inward flow to government for all university persona scenarios. Our modeling demonstrates the potential of increased return on investment that would outweigh initial investment in programs and allow for additional inward cash flow due to better employment.

**Figure 1:**
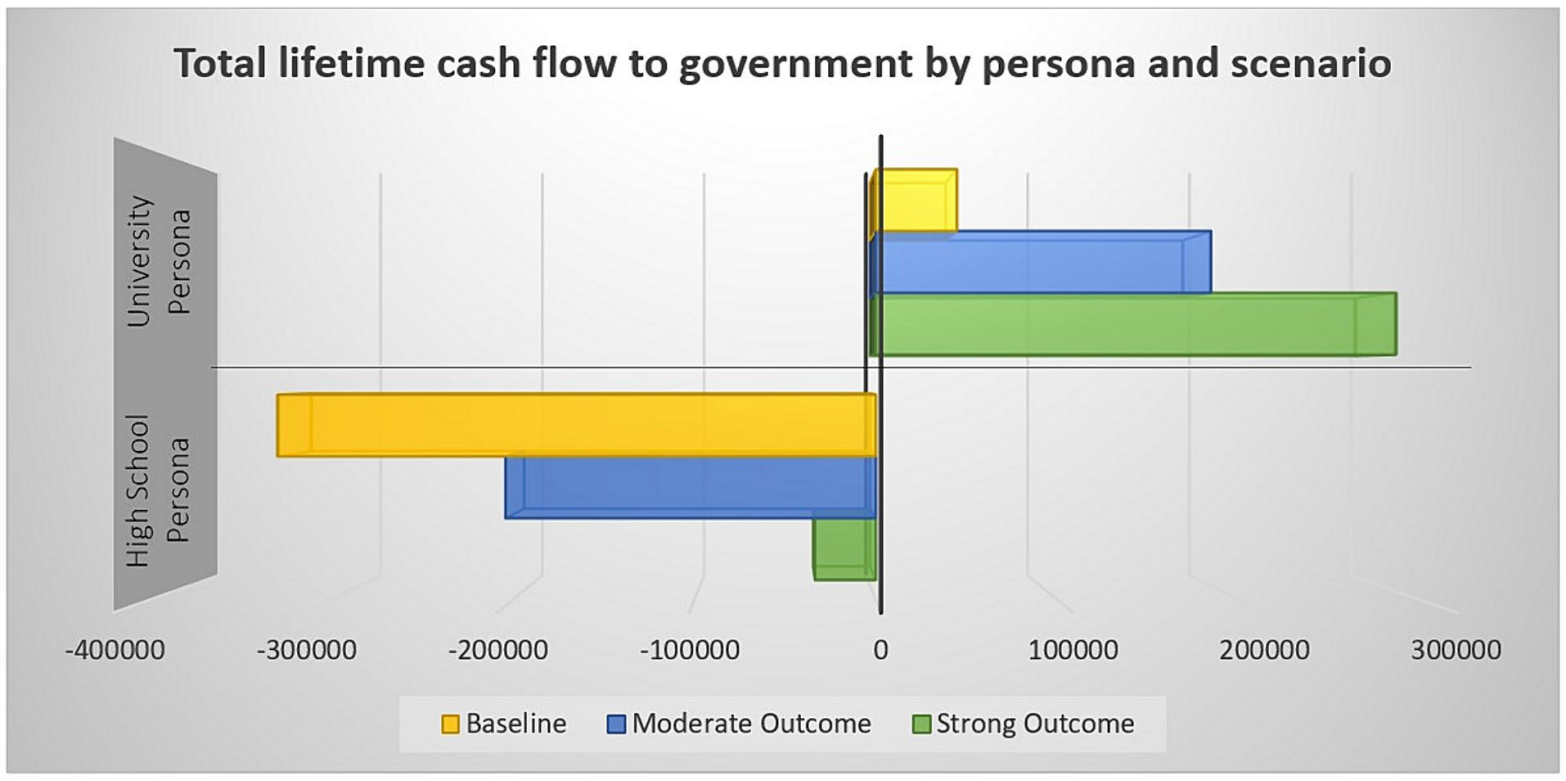
A bar graph comparing the total lifetime cash flow to government for each persona and scenario using the information provided in the results section. Graph indicates that money increasingly retained by or returned to government with more training as described in the scenarios.

As highlighted in Figure 1, intervention scenarios for both personas demonstrate higher government return (either reduced government payout or greater return to government via income tax revenue). These outcomes were modeled even with our attempts at conservative inputs.

To understand the sensitivity of our model to changes in the variables, four sensitivity analyses were run. We alternately varied the inflation rate, discount rate, inclusion of RRSP contributions (university persona only), and exclusion of the DTC. Full descriptions are available in Supplementary Appendix C. The four sensitivity analyses were chosen as most relevant to the topic and current economic climate. Due to our conservative approach to model construction, all sensitivity analyses supported increased lifetime cash flow to government as compared to their respective baseline scenarios. We note that any of the variables in the model can be altered to see the effect on the return, down to any of the five earnings variables for a specific year for a given persona-scenario combination.

## 4 Discussion

There is a swell of emerging interest and evidence supporting employment participation for people with disabilities in literature relating to disability rights, rehabilitation sciences, and economics. Given the potential for people with disabilities to diversify our labor force and reduce potential labor shortages, we identified investment in the best-practice of early employment participation (Kohler et al., 2017; National Collaborative on Workforce and Disability for Youth, 2018) as a realistic and meaningful step toward their successful labor force participation. Such labor force participation would benefit the individuals with disabilities, the organizations that employ them, and society more broadly.

To promote equitable and reliable access to the essential employment support services necessary for labor market participation, we suggest that early intervention programs be supported via public funding. Data on the current existence of such programs internationally, as well as their components and outcomes are currently limited. The data needed to support investment in such largescale programming is therefore unavailable. It will remain unavailable without public funding, and so we have opted to create our theoretically-driven cost–benefit model to project costs and public savings. In this paper, we provided examples of current Ontario-based programs being implemented, but do not promote one program or intervention model over others. The cost–benefit modeling was meant to represent the potential of investment in evidence-based employment support services that exist in the health and rehabilitation, education, community, social service, and private sectors.

The theoretical outputs will help us to begin the conversation on how to best publicly support this type of programming. This clear and present need for an evidence-based and data-centric approach aligns with the recent interim report of the final review of the Accessibility for Ontarians with Disabilities Act (Donovan, 2023). In this report, the reviewer highlights “a lack of data as a significant problem… it makes it impossible for organizations, especially municipalities to gauge success for accessibility solutions,” (p. 16). In our clinical and advocacy work, we see the difference that evidence-based early interventions can make, and simultaneously acknowledge the need for largescale data to implement province-wide initiatives. We therefore offer the modeling in this paper as a theoretical benchmark and set of evaluation metrics to be reviewed at a provincial level, or possibly at a pilot level to build a preliminary dataset for wider application within the province.

Our model allowed us to concurrently consider the best available evidence, current policies and funding structures, and intervention costs, all applied within a model that accommodates for inflation. Even when applied with the conservative parameters above, there are clear net benefits to public investment in early employment participation interventions for people with disabilities. The benefits are even more relevant when considered in the context of systems that *already* invest public funds into the daily life and care of adults with disabilities, as is the case in Ontario, Canada. The model was purposely designed to be adaptable to other funding structures in the future. Readers are encouraged to review the inputs outlined in Tables 1–3 and the Supplementary materials provided to consider how their public funding might be similarly assessed, such as places with aligning public funding models, like other Canadian provinces and territories.

To support the broader applicability or adaptability of our model outside of Ontario and Canada, our findings align with the small but growing literature specifically exploring public benefit of early employment investment. In their systematic review, Jacob et al. (2015) explored how employment services related to better employment outcomes for adults with Autism Spectrum Disorders, reducing government costs when compared with standard care or no intervention. Citing studies from the US, UK, and Sweden, the individual lifetime savings of reducing access to public care/insurance funds following employment exceed investments in effective employment support and training. In their work, public savings related to Social Security Insurance, public day programs and care, lost productivity that could be realized from adults with ASD who are able to engage productively but have not had the appropriate training and support. Calculated costs were related to providing vocational rehabilitation or employment training interventions.

Beyer (2017) reported on the economic impact of inclusion of people with disabilities in the labor market from a primarily European perspective. Based on his review of cost–benefit analyses from the perspective of tax-payers, Beyer concluded that society financially benefits from the availability of a variety of employment preparation interventions (e.g., supported employment, individual placement and support, vocational rehabilitation, social enterprise approach, and diversity placement approach). He noted that the evidence weighed heavily toward overall return on investment per dollar spent on such services for individuals with disabilities and taxpayers, with return on investment growing with longer-term engagement in employment, and that even when return on investment did not reach 1.0, gains were seen compared to no intervention. Given a major limitation to his findings were the disparity among data collection methods, model comparisons, and national contexts, we saw a need to create a fairly adaptable theoretical model to allow for calculation and comparison as presented in this paper.

The evidence on public benefit for early supported employment interventions is not limited to theoretical exploration or modeling. In a US study, Anderson et al. (2019) undertook a cost–benefit analysis of the Wisconsin PROMISE program, which supported transition-aged youth who receive public funding through Supplemental Security Income. PROMISE programs engage youth to achieve competitive, integrated employment. The team found that 67% of 14–18 year old youth receiving their employment support intervention reported at least some engagement with employment during the intervention period, compared to 57% of control participants. Projections of continued engagement with employment into adult years for these groups indicate a likelihood of youth who accessed vocational training services to earn wages at the Substantial Gainful Activity level, and eventually earn enough to work without supplemental security income benefits. The trajectory of greater earnings for these youth into adulthood is projected to lead to increased lifetime earnings and increased tax revenue following from an initial investment of employment preparation case management. In another US-based study, Schochet (2021) demonstrated that for the 20–24 year old participants in his study of the Job Corps employment support intervention, public costs invested per individual were expected to reach a breakeven point by participant age 50, at which time the amount that the individual contributed via taxes plus the amount saved via reduced public intervention compared to age-matched peers would exceed public investment. These findings indicate similar potential outcomes coming from various international social assistance, educational, and vocational support systems.

Based on the model’s application in this paper and the growing body of supporting evidence, we advocate for a shift in the public funding structure to include – and promote – employment training and supports upstream (i.e., in high school and young adulthood). Upstream investment in evidence-based programming is projected to lead to more people benefiting from intervention over longer periods. On a per-person basis, both presented scenarios demonstrate notable lifetime savings of public funding based on an early investment approach. The model was responsive to sensitivity analyses, none of which indicated that realistic alternate inputs would change the net benefit to society.

### 4.1 Limitations and future directions

Our model is not exhaustive. The most notable limitation is the assumption of effectiveness of the presented interventions (Wehman et al., 2020; Bowman et al., 2023), and the applicability of our constructed personas. As outlined in the introduction, the research-based evidence and our expert collaborators’ input indicate high correlation between early employment participation interventions and employment participation, particularly paid employment. We have modeled based on that assumption, but cannot guarantee outcomes for all participants. When applying our model at a population-level, there will never be 100% participation in the labor force following evidence-based interventions, just as that level of workforce participation does not exist in the general Canadian population.

A related limitation is that, while we discuss the effectiveness for the population of people with disabilities, we acknowledge this as a diverse group with varied profiles, characteristics, and support needs. We developed a model that we believe can be agile in capturing public flow of dollars per person based on parameters that are fairly widely utilized as they relate to disability, but know that we cannot fully capture this without further investigation. We propose this as an opportunity for future research to broaden our understanding of the impacts of supporting a variety of starting early approaches.

We chose not to include certain salient data points located between the individual and macrosystemic levels. Examples of the complex, interwoven nature of such variables can be seen in works such as that by Tompa et al. (2021). These variables extend beyond the scope of our current paper, and will add interesting and relevant information to future iterations of our modeling. Notable exclusions that are often described but not quantified include the role of unpaid caregivers who might be unable to work themselves due to their caregiving duties for those not engaged in the workforce (usually family members) and the costs and benefits to employers [e.g. additional training, accommodations, training on diversity, equity and inclusion (Beyer, 2017), lower staff turnover (Jacob et al., 2015)]. We did not specifically include the voices of employers, considerations of discriminatory hiring practices, or reluctance or stigma related to hiring employees with disabilities as they do not directly relate to the cost–benefit model outlined in this paper, but we acknowledge their presence and weight regarding inclusive hiring. While not formally included in the cost–benefit modeling, the literature available surrounding such elements would still appear to weigh in favor of the societal-level benefits of funding early employment participation interventions that would increase practice, upskilling, and early labor market attachment.

We also acknowledge our inability to fully capture and quantify the non-financial benefits of employment for a person, their family, community and society. Examples include socializing and friendship, community participation, mental and physical health and engagement in occupations that help one work toward fulfillment and self-actualization. These benefits are not represented in our model, but must be considered in both future financial calculations (e.g., Tompa et al., 2021), as well as for their inherent value of facilitating the movement of citizens into meaningful community engagement.

It is beyond the scope of this paper to comment on how the funding would be approved and distributed, as well as the types of organizations that would oversee the interventions. In our examples, the Project SEARCH program is jointly run through multiple organizations, including school boards and employment support agencies (representing multiple ministries and sectors). The Employment Pathways programs were run through a pediatric rehabilitation hospital. The programs highlighted in our discussion were run through various agencies, both public and private, youth-and adult-oriented.

Future exploration can model *how* funds will be shifted upstream in order to intervene earlier in the lifespan when proposed savings would not be realized for years. As previously mentioned, it is also important to explore the applicability of the model to broader populations with disabilities entering the workforce, which requires identification of appropriate interventions and consideration of other potential parameters within the model. We also recommend studies exploring the possible and optimal agencies for delivering publicly funded early employment support interventions. More research is also required to solidify the types of interventions, quality parameters, and optimal delivery formats that would allow for the realization of the theoretical societal benefits proposed by our model. Additional parameters of acceptable outcomes, such as participation in post-secondary education or training, job quality, types of benefits and stability offered by the employer, will also be important to consider when determining “success” of funded interventions (Caliendo and Schmidl, 2016). We also recommend exploring the role of employers in early employment engagement for individuals with disabilities, including factors that may support or hinder their engagement in hiring and supporting growth.

## 5 Conclusion

Our cost–benefit model presents a case for investing in early employment participation intervention for youth with disabilities, using Ontario, Canada as an exemplar. By applying the best available evidence and practices in the area of start-early employment participation for youth with disabilities, together with expert input, our model projects that public investment in early employment intervention will demonstrate societal-level benefits per person served. Based on our two personas, the theoretical lifetime societal-level impact of investment in intervention for a person with disabilities will lead to long-term public savings, in addition to the important individual, community, corporate, labor market, and cultural impacts that employment and inclusion demonstrate. Having a model for funding interventions represents an important early step in increasing the reliable domestic labor pool and moving toward more equitable job markets.

## Data availability statement

The original contributions presented in the study are included in the article/Supplementary material, further inquiries can be directed to the corresponding author.

## Ethics statement

Ethical approval was not required for the studies involving humans in accordance with the local legislation and institutional requirements. The participants provided their written informed consent for participation in the knowledge mobilization project, and consented to having the aggregated results of discussions shared through outputs such as academic papers.

## Author contributions

LB: Conceptualization, Investigation, Methodology, Project administration, Resources, Writing – original draft, Writing – review & editing. CM: Conceptualization, Formal analysis, Investigation, Project administration, Writing – original draft, Writing – review & editing. RD: Data curation, Formal analysis, Investigation, Methodology, Validation, Writing – original draft, Writing – review & editing. BP: Conceptualization, Data curation, Investigation, Validation, Writing – review & editing. YX: Data curation, Investigation, Writing – original draft. JC: Conceptualization, Validation, Writing – review & editing.

## Footnotes

^1^Ontario’s new milestone payment model under the 2020 “Employment Services Transformation” is currently in effect in a pilot phase in 12 of Ontario’s 16 catchment areas. We constructed our model using the original model still active in 4 catchment areas, which was the predominant model at the time of manuscript production. This choice aligns with the conservative estimate of a net benefit, as the new payments may be higher on a per-individual basis.

^2^Program cost includes salaries and benefits for 2 skills trainers at average Ontario wages. A teacher also supports the program, assigned and paid by the participating school board. Because the teacher’s salary would be incurred regardless of the type of school programming (typical classroom or Project SEARCH), it is therefore not reflected.

## Supplementary material

Supplementary Appendix A Full description of model construction.

Supplementary Appendix B Full earnings variables values for all personas/scenarios.

Supplementary Appendix C Full description.
