# Supplementary material for: Funding employment inclusion for Ontario youth with disabilities: a theoretical cost-benefit model
Source: Front Sociol. 2024 Apr 10;9:1281088. doi: 10.3389/fsoc.2024.1281088 (PMC11041630; doi:10.3389/fsoc.2024.1281088)
Supplement: Supplementary Appendix A — Full description of model construction. [file Data_Sheet_1.PDF]

Funding Employment Inclusion for Ontario Youth with Disabilities: A Cost-Benefit Model

Bowman, McDougall, Doucet, Pooran, Xu & Campbell

Supplementary Appendix A. Full description of model construction

A.1 Calculating the Return on the Intervention

In keeping with the Treasury Board of Canada's Cost Benefit Analysis Guide (2007), we establish a baseline scenario and compare the intervention-specific scenarios with the baseline scenario to calculate the relative net benefits. We calculate the net present value (NPV) of the cash flows of each scenario to obtain the relative net benefits and then divide the result by the cost of the intervention to produce a return, as described in the following formula.

$$Return = \frac{NPV\ CF\ Program\ Scenario - NPV\ CF\ Baseline\ Scenario}{PV\ Program\ Cost}$$

In our model, we look strictly at the costs and benefits accruing to government, not to business or hypothetical individuals accessing programs. In theory, this will yield a conservative return, as the model accounts for all of the program costs but does not account for all the benefits – both direct and indirect – experienced by individuals and businesses. As is seen in the formula above, we include the single cost of delivering the intervention. This is the only cost the government would be incurring (through funding issued to the provider of the intervention). Given that our baseline and intervention scenarios will always use an equivalent duration, we do not annualize the present value and thus use the overall NPV when calculating the net benefits (Firms Consulting, 2010). Our formula is presented in Table 1 of the main document, and reiterated below.

$$NPV = \sum_{n=0}^{N} \frac{(IAIT_n - IATC_n) - (ODSPES_n + ODSPIS_n)}{(1 + r_n)^n}$$

We employ the widely-used discounted cash flow (DCF) analysis, whereby costs and benefits for a given scenario are projected out into the future and then discounted back to present day dollars. We identified the monetary benefits to government based on the fact that an individual's extra lifetime earnings resulting from their participation in an intervention (compared to the baseline scenario) would be taxable and would thus generate revenue, the amount for which can be derived from published formulas in the tax code. This is undertaken with the acknowledgement that for net tax revenue to occur, extra earnings will need to be high enough to avoid being offset by unused deductions or tax credits that would apply in the baseline scenario. In addition to taxable earnings, social assistance for persons with disabilities (e.g. ODSP Income Supports, Figure A.2) is a function of income levels and so the *avoided* social assistance paid out resulting from higher earnings can also be derived using published formulas.

We thus identify the benefits that can be derived from annual earnings using published formulas, grouping them into two categories: *a) tax revenue* and *b) avoided social assistance costs*. Using the DCF method mentioned above, we designed the model to calculate the NPV of tax revenue and social assistance costs for each year of an N-year stream of projected annual earnings under the baseline scenario and one or more intervention scenarios. We show this by elaborating the formula for calculating the NPV of the relevant cash flow streams *to government* for a single scenario.

We further adjusted for inflation in the model for those inputs inside the tax revenue and social assistance calculations that can reasonably be assumed to increase over time to keep pace with the cost of living, such as tax bracket thresholds and social assistance income thresholds. Those factors are available in Table A2 (see values marked "Inflation-Adjusted" in the column "Subject to Increase/Decrease Over Time"). For the purposes of model clarity, we have chosen

to express all dollar values in nominal flows (i.e. after having factored in inflation) and, therefore, to discount them back using nominal rates.

A.2 Model Inputs

We identify three groups of model inputs, based on their nature and scope (Table 2 in the main text and A1, A2 in this document). The groups are “Global”, “Local Context” and “Persona”. *Global inputs* are values that would remain consistent across contexts, namely the inflation and discount rates. *Local context inputs* represent the local tax regime (“Tax”), social assistance programs (“SA”) and labour market (“LM”). These include tax brackets, tax thresholds, tax credits, tax rates, social assistance income thresholds, social assistance payments and minimum wage. In its initial implementation (the current paper), the model uses the Ontario, Canada context. *Persona inputs* are those used to define a persona-scenario and are further broken into two subgroups: annual earnings over a working lifetime (“Earnings”) and possible tax strategies (“Tax Strategies”). Given that the target demographic of our model (i.e. low attachment to the labour market) will likely be working wage-based jobs, we include the following inputs: working years, weeks per year, hours per week, starting wage, and wage progression. The model can also accommodate annual salaries, whereby the salary replaces the combination of weeks per year, hours per week and wage.

Table A1. Summary of Model Input Types

**Input Type**	**Example Category**
Global	Inflation rate, discount rate
Local Context	Tax brackets, tax thresholds, tax credits, tax rates, social assistance income thresholds, social assistance payments, minimum wage
Persona – Earnings	Lifetime working years, weeks per year, hours per week, starting wage, wage progression
Persona – Tax Strategy	Registered savings plans contributions, disability-related deductions, disability tax credits claimed

Table A2. Expanded List of Model Input by Type

	Input	Source	Subject to Increase/ Decrease Over Time
Global	Inflation rate (2.2%)	https://www.bankofcanada.ca/core-functions/monetary-policy/inflation/	N/A
	Discount rate used (SDR = 3.0%)	Treasury Board of Canada (2007)	N/A
Local Context	Minimum wage	Government of Ontario (2017)	Yes, inflation-adjusted
	Level-2 wage	Statistics Canada (2022)	Yes, inflation-adjusted
	EI Max Earnings	https://www.canada.ca/en/revenue-agency/services/tax/businesses/topics/payroll/payroll-deductions-contributions/employment-insurance-ei/ei-premium-rates-maximums.html	Yes, inflation-adjusted
	EI Contribution Rate	https://www.canada.ca/en/revenue-agency/services/tax/businesses/topics/payroll/payroll-deductions-contributions/employment-insurance-ei/ei-premium-rates-maximums.html	Yes, inflation-adjusted
	Federal and Ontario income tax **rates**	https://www.canada.ca/en/services/taxes/income-tax.html	No
	Federal and Ontario income tax **brackets**	https://www.canada.ca/en/services/taxes/income-tax.html	Yes, inflation-adjusted
	Disability tax credit	https://www.canada.ca/en/revenue-agency/services/tax/individuals/segments/tax-credits-deductions-persons-disabilities/disability-tax-credit.html	Yes, inflation-adjusted
	Program Cost	Expert input	N/A (all costs incurred in year 1 or 2)
	ODSP ES Milestone Cost	https://www.ontario.ca/document/ontario-disability-support-program-policy-directives-employment-supports/51-employment	No
	ODSP ES Retention Months	https://www.ontario.ca/document/ontario-disability-support-program-policy-directives-employment-supports/51-employment	No
	ODSP IS Exemption	https://www.ontario.ca/document/ontario-disability-support-program-policy-directives-income-support/51-definition-and	No

	ODSP IS Disability-related Expenses	https://www.ontario.ca/document/ontario-disability-support-program-policy-directives-income-support/51-definition-and	Yes, inflation-adjusted
	ODSP IS Childcare-related Expenses	https://www.ontario.ca/document/ontario-disability-support-program-policy-directives-income-support/51-definition-and	Yes, inflation-adjusted
	ODSP IS Clawback rate	https://www.ontario.ca/page/working-and-earning-ontario-disability-support-program	No
	ODSP Base Amount (Monthly)	https://www.ontario.ca/page/working-and-earning-ontario-disability-support-program	Yes, 1% increase
	ODSP Extended Health, Dental and Vision amount	https://www.ontario.ca/page/ontario-disability-support-program-health-and-disability-benefits	Yes, inflation-adjusted
Persona: Tax Strategy	CPP Max Earnings	https://www.canada.ca/en/revenue-agency/services/tax/businesses/topics/payroll/payroll-deductions-contributions/canada-pension-plan-cpp/cpp-contribution-rates-maximums-exemptions.html	Yes, inflation-adjusted
	CPP Contribution Rate	https://www.canada.ca/en/revenue-agency/services/tax/businesses/topics/payroll/payroll-deductions-contributions/canada-pension-plan-cpp/cpp-contribution-rates-maximums-exemptions.html	No
	RRSP Contribution	Persona/scenario	No
	Disability-related employment expenses	Persona/scenario	Yes, inflation-adjusted
	Childcare	Persona/scenario	Yes, inflation-adjusted
	Federal and Ontario basic deduction	Persona/scenario	Yes, inflation-adjusted
Persona: Earnings	Number of working years	Persona/scenario	N/A
	Hours/week	Persona/scenario	N/A
	Wage/salary	Persona/scenario	Yes, inflation-adjusted
	Working weeks per year	Persona/scenario	N/A
	Alternate Community Supports	Persona/scenario	Yes, inflation-adjusted

	CPP Basic Exemption	https://www.canada.ca/en/revenue-agency/services/tax/businesses/topics/payroll/payroll-deductions-contributions/canada-pension-plan-cpp/cpp-contribution-rates-maximums-exemptions.html	Yes, inflation-adjusted

A.3 Tax Calculation Engine

We determined the scope of the tax calculation engine based on what is needed to produce accurate outputs (refund or balance owing) for *a single individual at any income level*, and who:

1. may or may not claim disability-related tax credits
2. may or may not have registered savings contributions, such as a registered retirement savings plan (RRSP)
3. may or may not have disability-related employment expenses

The engine outputs tax revenue (positive or negative) for those individuals that meet the criteria described above, including the three optional criteria. They are represented as Inflation Adjusted Income Tax (IAIT) (inflation-adjusted income tax owed) and Inflation Adjusted Tax Credits (IATC) (inflation adjusted amount owed back to the individual) in the formula in main document Table 1.

Given employment earnings of an individual, with or without the tax-relevant credits and contributions listed, we designed the model to generate the net tax generated for any given year. Table A3 provides information on the outputs used within the Ontario, Canada context that were generated by our model, and subsequently used to produce the net tax generated for a given year. We subsequently tested our model against results from the 2021 version of ProFile (a Canada Revenue Agency-authorized tax software package) at multiple income levels to confirm the accuracy of our calculations across scenarios.

Table A3. Tax-related Outputs Generated by Our Model for the Ontario Context

**Tax Level**	**Item**
Pension and Employment Insurance	Canada Pension Plan<br>Employment Insurance
Federal Tax	Federal Non-Refundable Tax Credits<br>Federal Refundable Tax Credits<br>**Federal Tax Owing/Refund**
Provincial (Ontario) Tax	Ontario Refundable Tax Credits<br>Ontario Non-Refundable Tax Credits<br>Ontario Surtax<br>Ontario LIFT (Low-income Individuals and Families Tax credit)<br>Ontario Health Premium<br>**Ontario Tax Owing/Refund**

Two forms of revenue related to social assistance and support were excluded from our tax calculations because they result in lifetime net of zero dollars. The first is revenue from the Ontario Disability Support Program's (ODSP) income supports (Ontario, 2021b) as it results in net zero dollars, by definition (it is considered as income on the tax return (T1) but later subtracted before taxable income is calculated). The second is Canada Pension Plan Disability (CPP-D), which, by definition, is only paid out if the person in question is unable to work. Given that our model assumes the personas under all scenarios are employed throughout their working lifetime, we disregard the CPP-D.

We also highlight that, in practice, other possible tax deductions could apply to an individual in a given year. Due to their situational nature and sheer volume, we excluded other possible tax deductions from the tax calculation of our model. We factored in both standard and disability-specific deductions, and therefore assume that the model accounts for the primary – and the majority of – deductions for an individual in the target population. We do not currently support more complex tax scenarios such as multi-person households or situation-specific deductions (e.g. tuition, medical expenses). For the latter, these additional deductions would only impact the results if the additional tax revenue generated by the intervention scenario as

compared to the baseline scenario were disproportionately offset by the additional deductions. This might occur when the tax revenue is already zero for a given year in the baseline scenario, rendering the additional deductions meaningless, whereas in the intervention scenario the additional deductions serve to decrease or nullify the positive tax revenue. The model was designed to provide an overview of the individuals and scenarios encountered by our stakeholder group, and to represent conservative accounts of the impacts of intervention. Being extensible, the model could be updated in a future version to account for one or more additional deductions.

Finally, we recognize that certain inputs to the tax calculation are adjusted regularly (either yearly or at intervals of 2-5 years), and we adjust these inputs for inflation, keeping in mind that the inflation rate is itself an input to the model. In general, we assume that in order to preserve the intended effect of tax policy, tax rates *remain constant* over time whereas income thresholds, tax credits and payments *rise with inflation*. A number of factors at all levels of taxation have been inflation-adjusted in the model, as outlined in Table A2.

A.4 Social Assistance Calculation Engine

Social assistance costs must be considered in regionally-specific ways for readers outside of Ontario. Our social assistance costs, in the form of ODSP Employment Supports and Income Supports, were obtained from the relevant Ontario government websites (Ontario Ministry of Children, n.d.). We assume in our scenarios that *all available* ODSP employment and income supports would be claimed if an employer and/or individual was eligible to receive them. We thus built a social assistance engine that calculates both ODSP amounts paid out by the province, which represent a cost to government: employment supports (paid to service providers) and income supports (paid to individuals). Both of the ODSP amounts are a function of an individual's annual earnings, despite sometimes needing to account for eligible deductions.

Social assistance thresholds are determined by policy and should, in theory, be adjusted to reflect inflation. In practice, thresholds are adjusted much less frequently than yearly. For example, the amount paid to ODSP Employment Service providers for the 6- and 13-week milestones is not adjusted for inflation and has instead remained at $1,000 and $6,000, respectively, since at least 2012. We have adjusted those amounts to account for inflation when they have either historically risen with inflation or there is an official policy that signals the intent.

ODSP employment supports are the amounts paid by the government to a service provider that has placed an individual into employment and/or helped them retain the employment, according to a milestone payment schedule. We note that we used the old Ontario milestone payment model, which remains in effect in 13 of the province's 16 catchment areas, and have not updated to the new one currently being piloted under the 2020 "Employment Services Transformation." We assume conservatively that personas in the intervention scenarios outlined later will make full use of ODSP payments, even though they may have already transitioned to employment without the need of service providers, due to having been involved in the intervention. The detailed Employment Supports calculation is included as Figure A1. We calculated retention payments using 60% of monthly chargeable earnings for all 33 months, which is a minor simplification of this amount actually being paid at $250 minimum *or* 60% of chargeable earnings, whichever is greater. Our simplification is conservative, as the net return on the program would be slightly higher if accounting for the $250 minimum (Government of Ontario, 2021a).

ODSP Employment Support payments are calculated as follows:

ODSP Employment Supports Cost = Placement Milestone + Monthly Retention

ODSP Employment Supports Cost = 6-wk Milestone + 13-wk Milestone
+ 33 months x Monthly Payment

ODSP Employment Supports Cost = $1,000 + $6,000
+ 33 months x (greater of $250 or 60% of chargeable earnings, where chargeable earnings is 50% of the total earnings after subtracting CPP, EI and taxes)

Source: Government of Ontario, 2021, "Employment supports funding"

Figure A1. Detailed ODSP Employment Supports Calculation

We also conservatively assume that each individual, regardless of the persona used, will be single, have no dependents, and be receiving food and shelter from a parent or other supportive person. The implication of this assumption is that the individual is receiving the lowest possible amount of income supports, which is the "Board and Lodging" amount for an individual with no spouse or dependents. In practice, we support independence, autonomy, and active social engagement for individuals with disabilities, but the assumption of living with caregivers was made to support the most simplified and conservative presentation of our model in terms of tax calculation. Other living conditions would likely imply increased income and other credits that would serve to demonstrate even higher returns within the model, and can be considered in future model iterations. The detailed calculation for Income Supports is available in Figure A2. We note that the calculation and amount of support was changed recently (November 2022) and our model reflects the newly proposed amounts.

ODSP Income Supports are calculated using the formula specified in O. Reg 222/98 s.38 (Treatment of Earnings), whereby

ODSP Income Supports Cost = ODSP Base Amount – Chargeable Earnings + Work Incentive

where:
Monthly Chargeable Earnings = Gross Earnings – Payroll Deductions (CPP, EI, Income Tax)
– $200 Flat Fee Exemption – 50% of Earnings After Exemption
– Monthly Child Care Expenses
– Disability-related Employment Expenses

*For simplicity, we ignore child support payroll deduction and wage garnishment, as these are very rarely claimed in practice

Sources: Government of Ontario, 2021, "Definition and treatment of income"
Government of Ontario, 2021, "Deductions from employment and training income"

Figure A2. Detailed ODSP Income Supports Calculation

Similar to the tax-related inputs, we adjust certain ODSP inputs for inflation. We note that several payment amounts and threshold amounts that could be reasonably expected to rise with inflation have not historically, particularly Employment Supports. In September 2022, the government of Ontario announced that, after several years of not increasing the Income Supports payment (which is $825 if using the Board and Lodging amount), it will be increasing the amount by 5%. We translate that into a cost-of-living adjustment, and therefore consider the amount to rise with inflation going forward, even if the increase is not applied every year but every few years. We therefore use inflation-adjustment in our model for Income Supports in terms of Base Exception Amounts and Payment Amounts. These represent a subset of the list of inputs found in Table A2.

References

Firms Consulting. (2010). Annualized Net Present Value (ANPV). *Blog B School* [Online]. Available from: https://blogbschool.com/2010/10/27/annualized-net-present-value-anpv/.

Government of Ontario. (2017). Minimum Wage. *Your guide to the Employment Standards Act.*

Government of Ontario. (2021a). *ODSP Employment Supports: Employment Supports Funding* [Online]. Available: https://www.ontario.ca/document/ontario-disability-support-program-policy-directives-employment-supports/51-employment [Accessed].

Government of Ontario. (2021b). *ODSP Income Supports: Definition and treatment of income* [Online]. Available: https://www.ontario.ca/document/ontario-disability-support-program-policy-directives-income-support/51-definition-and [Accessed].

Ontario Ministry Of Children, Community, and Social Services. (n.d.) Ontario Disability Support Program (ODSP): What is ODSP? Available: https://www.mcss.gov.on.ca/documents/en/mcss/social/info_sheets/ODSP_what.pdf.

Statistics Canada. (2022). Table 14-10-0064-01 Employee wages by industry, annual. *Data.* Ottawa, Canada: Statistics Canada.

Treasury Board of Canada. (2007). Canadian Cost-Benefit Analysis Guide: Regulatory Proposals *In:* SECRETARIAT, T. B. O. C. (ed.). Canada: Her Majesty the Queen in Right of Canada.
