# Supplementary material for: Funding employment inclusion for Ontario youth with disabilities: a theoretical cost-benefit model
Source: Front Sociol. 2024 Apr 10;9:1281088. doi: 10.3389/fsoc.2024.1281088 (PMC11041630; doi:10.3389/fsoc.2024.1281088)
Supplement: Supplementary Appendix B — Full earnings variables values for all personas/scenarios. [file Data_Sheet_2.PDF]

Supplementary Appendix B: Full Earnings Variables Values for All Personas/Scenarios

EARNINGS VARIABLES VALUES

Persona	Scenario	Year	Age	Inflation	Discount	Wks/Yr	Hrs /Wk	Wage Type	Nominal Wage or Salary	Last Year Nominal Wage or Salary	Wage Progression	Continue from Previous Year	Progressed Wage	Progressed Wage with Inflation
High School	Baseline	1	21	2.2%	3.0%	-	20	MW	15.50	-	0.25%	TRUE	15.50	15.50
High School	Baseline	2	22	2.2%	3.0%	48	20	MW	15.50	15.50	0.25%	TRUE	15.54	15.88
High School	Baseline	3	23	2.2%	3.0%	48	20	MW	15.50	15.54	0.25%	TRUE	15.58	16.27
High School	Baseline	4	24	2.2%	3.0%	48	20	MW	15.50	15.58	0.25%	TRUE	15.62	16.67
High School	Baseline	5	25	2.2%	3.0%	48	20	MW	15.50	15.62	0.25%	TRUE	15.66	17.08
High School	Baseline	6	26	2.2%	3.0%	48	20	MW	15.50	15.66	0.25%	TRUE	15.69	17.50
High School	Baseline	7	27	2.2%	3.0%	48	20	MW	15.50	15.69	0.25%	TRUE	15.73	17.93
High School	Baseline	8	28	2.2%	3.0%	48	20	MW	15.50	15.73	0.25%	TRUE	15.77	18.37
High School	Baseline	9	29	2.2%	3.0%	48	20	MW	15.50	15.77	0.25%	TRUE	15.81	18.82
High School	Baseline	10	30	2.2%	3.0%	48	20	MW	15.50	15.81	0.25%	TRUE	15.85	19.28
High School	Baseline	11	31	2.2%	3.0%	48	20	MW	15.50	15.85	0.25%	TRUE	15.89	19.76
High School	Baseline	12	32	2.2%	3.0%	48	20	MW	15.50	15.89	0.25%	TRUE	15.93	20.24
High School	Baseline	13	33	2.2%	3.0%	48	20	MW	15.50	15.93	0.25%	TRUE	15.97	20.74
High School	Baseline	14	34	2.2%	3.0%	48	20	MW	15.50	15.97	0.25%	TRUE	16.01	21.25
High School	Baseline	15	35	2.2%	3.0%	48	20	MW	15.50	16.01	0.25%	TRUE	16.05	21.77
High School	Baseline	16	36	2.2%	3.0%	48	20	MW	15.50	16.05	0.25%	TRUE	16.09	22.30
High School	Baseline	17	37	2.2%	3.0%	48	20	MW	15.50	16.09	0.25%	TRUE	16.13	22.85
High School	Baseline	18	38	2.2%	3.0%	48	20	MW	15.50	16.13	0.25%	TRUE	16.17	23.41
High School	Baseline	19	39	2.2%	3.0%	48	20	MW	15.50	16.17	0.25%	TRUE	16.21	23.99
High School	Baseline	20	40	2.2%	3.0%	48	20	MW	15.50	16.21	0.25%	TRUE	16.25	24.58
High School	Baseline	21	41	2.2%	3.0%	48	20	MW	15.50	16.25	0.25%	TRUE	16.29	25.18
High School	Baseline	22	42	2.2%	3.0%	48	20	MW	15.50	16.29	0.25%	TRUE	16.33	25.80
High School	Baseline	23	43	2.2%	3.0%	48	20	MW	15.50	16.33	0.25%	TRUE	16.38	26.43
High School	Baseline	24	44	2.2%	3.0%	48	20	MW	15.50	16.38	0.25%	TRUE	16.42	27.08
High School	Baseline	25	45	2.2%	3.0%	48	20	MW	15.50	16.42	0.25%	TRUE	16.46	27.74
High School	Baseline	26	46	2.2%	3.0%	48	20	MW	15.50	16.46	0.25%	TRUE	16.50	28.43
High School	Baseline	27	47	2.2%	3.0%	48	20	MW	15.50	16.50	0.25%	TRUE	16.54	29.12
High School	Baseline	28	48	2.2%	3.0%	48	20	MW	15.50	16.54	0.25%	TRUE	16.58	29.84
High School	Baseline	29	49	2.2%	3.0%	48	20	MW	15.50	16.58	0.25%	TRUE	16.62	30.57
High School	Baseline	30	50	2.2%	3.0%	48	20	MW	15.50	16.62	0.25%	TRUE	16.66	31.32
High School	Baseline	31	51	2.2%	3.0%	48	20	MW	15.50	16.66	0.25%	TRUE	16.71	32.09
High School	Baseline	32	52	2.2%	3.0%	48	20	MW	15.50	16.71	0.25%	TRUE	16.75	32.88
High School	Baseline	33	53	2.2%	3.0%	48	20	MW	15.50	16.75	0.25%	TRUE	16.79	33.69
High School	Baseline	34	54	2.2%	3.0%	48	20	MW	15.50	16.79	0.25%	TRUE	16.83	34.51
High School	Baseline	35	55	2.2%	3.0%	48	20	MW	15.50	16.83	0.25%	TRUE	16.87	35.36
High School	Baseline	36	56	2.2%	3.0%	48	20	MW	15.50	16.87	0.25%	TRUE	16.92	36.23
High School	Baseline	37	57	2.2%	3.0%	48	20	MW	15.50	16.92	0.25%	TRUE	16.96	37.12
High School	Baseline	38	58	2.2%	3.0%	48	20	MW	15.50	16.96	0.25%	TRUE	17.00	38.03
High School	Baseline	39	59	2.2%	3.0%	48	20	MW	15.50	17.00	0.25%	TRUE	17.04	38.96
High School	Baseline	40	60	2.2%	3.0%	48	20	MW	15.50	17.04	0.25%	TRUE	17.09	39.92
High School	Baseline	41	61	2.2%	3.0%	48	20	MW	15.50	17.09	0.25%	TRUE	17.13	40.90
High School	Baseline	42	62	2.2%	3.0%	48	20	MW	15.50	17.13	0.25%	TRUE	17.17	41.91
High School	Baseline	43	63	2.2%	3.0%	48	20	MW	15.50	17.17	0.25%	TRUE	17.21	42.94
High School	Baseline	44	64	2.2%	3.0%	48	20	MW	15.50	17.21	0.25%	TRUE	17.26	43.99
High School	Baseline	45	65	2.2%	3.0%	48	20	MW	15.50	17.26	0.25%	TRUE	17.30	45.07
High School	Moderate	1	21	2.2%	3.0%	24	24	S2	17.35	-	0.25%	TRUE	17.35	17.35
High School	Moderate	2	22	2.2%	3.0%	48	24	S2	17.35	17.35	0.25%	TRUE	17.39	17.78
High School	Moderate	3	23	2.2%	3.0%	48	24	S2	17.35	17.39	0.25%	TRUE	17.44	18.21
High School	Moderate	4	24	2.2%	3.0%	48	24	S2	17.35	17.44	0.25%	TRUE	17.48	18.66
High School	Moderate	5	25	2.2%	3.0%	48	24	S2	17.35	17.48	0.25%	TRUE	17.52	19.12
High School	Moderate	6	26	2.2%	3.0%	48	24	S2	17.35	17.52	0.25%	TRUE	17.57	19.59
High School	Moderate	7	27	2.2%	3.0%	48	24	S2	17.35	17.57	0.25%	TRUE	17.61	20.07
High School	Moderate	8	28	2.2%	3.0%	48	24	S2	17.35	17.61	0.25%	TRUE	17.66	20.56
High School	Moderate	9	29	2.2%	3.0%	48	24	S2	17.35	17.66	0.25%	TRUE	17.70	21.07
High School	Moderate	10	30	2.2%	3.0%	48	24	S2	17.35	17.70	0.25%	TRUE	17.74	21.58
High School	Moderate	11	31	2.2%	3.0%	48	24	S2	17.35	17.74	0.25%	TRUE	17.79	22.11
High School	Moderate	12	32	2.2%	3.0%	48	24	S2	17.35	17.79	0.25%	TRUE	17.83	22.66
High School	Moderate	13	33	2.2%	3.0%	48	24	S2	17.35	17.83	0.25%	TRUE	17.88	23.21
High School	Moderate	14	34	2.2%	3.0%	48	24	S2	17.35	17.88	0.25%	TRUE	17.92	23.78
High School	Moderate	15	35	2.2%	3.0%	48	24	S2	17.35	17.92	0.25%	TRUE	17.97	24.37
High School	Moderate	16	36	2.2%	3.0%	48	24	S2	17.35	17.97	0.25%	TRUE	18.01	24.96
High School	Moderate	17	37	2.2%	3.0%	48	24	S2	17.35	18.01	0.25%	TRUE	18.06	25.58
High School	Moderate	18	38	2.2%	3.0%	48	24	S2	17.35	18.06	0.25%	TRUE	18.10	26.21
High School	Moderate	19	39	2.2%	3.0%	48	24	S2	17.35	18.10	0.25%	TRUE	18.15	26.85
High School	Moderate	20	40	2.2%	3.0%	48	24	S2	17.35	18.15	0.25%	TRUE	18.19	27.51
High School	Moderate	21	41	2.2%	3.0%	48	24	S2	17.35	18.19	0.25%	TRUE	18.24	28.18
High School	Moderate	22	42	2.2%	3.0%	48	24	S2	17.35	18.24	0.25%	TRUE	18.28	28.88
High School	Moderate	23	43	2.2%	3.0%	48	24	S2	17.35	18.28	0.25%	TRUE	18.33	29.59
High School	Moderate	24	44	2.2%	3.0%	48	24	S2	17.35	18.33	0.25%	TRUE	18.38	30.31
High School	Moderate	25	45	2.2%	3.0%	48	24	S2	17.35	18.38	0.25%	TRUE	18.42	31.06
High School	Moderate	26	46	2.2%	3.0%	48	24	S2	17.35	18.42	0.25%	TRUE	18.47	31.82
High School	Moderate	27	47	2.2%	3.0%	48	24	S2	17.35	18.47	0.25%	TRUE	18.51	32.60
High School	Moderate	28	48	2.2%	3.0%	48	24	S2	17.35	18.51	0.25%	TRUE	18.56	33.40

EARNINGS VARIABLES VALUES

Persona	Scenario	Year	Age	Inflation	Discount	Wks/Yr	Hrs /Wk	Wage Type	Nominal Wage or Salary	Last Year Nominal Wage or Salary	Wage Progression	Continue from Previous Year	Progressed Wage	Progressed Wage with Inflation
High School	Moderate	29	49	2.2%	3.0%	48	24	S2	17.35	18.56	0.25%	TRUE	18.61	34.22
High School	Moderate	30	50	2.2%	3.0%	48	24	S2	17.35	18.61	0.25%	TRUE	18.65	35.06
High School	Moderate	31	51	2.2%	3.0%	48	24	S2	17.35	18.65	0.25%	TRUE	18.70	35.92
High School	Moderate	32	52	2.2%	3.0%	48	24	S2	17.35	18.70	0.25%	TRUE	18.75	36.80
High School	Moderate	33	53	2.2%	3.0%	48	24	S2	17.35	18.75	0.25%	TRUE	18.79	37.71
High School	Moderate	34	54	2.2%	3.0%	48	24	S2	17.35	18.79	0.25%	TRUE	18.84	38.63
High School	Moderate	35	55	2.2%	3.0%	48	24	S2	17.35	18.84	0.25%	TRUE	18.89	39.58
High School	Moderate	36	56	2.2%	3.0%	48	24	S2	17.35	18.89	0.25%	TRUE	18.93	40.55
High School	Moderate	37	57	2.2%	3.0%	48	24	S2	17.35	18.93	0.25%	TRUE	18.98	41.55
High School	Moderate	38	58	2.2%	3.0%	48	24	S2	17.35	18.98	0.25%	TRUE	19.03	42.57
High School	Moderate	39	59	2.2%	3.0%	48	24	S2	17.35	19.03	0.25%	TRUE	19.08	43.62
High School	Moderate	40	60	2.2%	3.0%	48	24	S2	17.35	19.08	0.25%	TRUE	19.12	44.69
High School	Moderate	41	61	2.2%	3.0%	48	24	S2	17.35	19.12	0.25%	TRUE	19.17	45.78
High School	Moderate	42	62	2.2%	3.0%	48	24	S2	17.35	19.17	0.25%	TRUE	19.22	46.91
High School	Moderate	43	63	2.2%	3.0%	48	24	S2	17.35	19.22	0.25%	TRUE	19.27	48.06
High School	Moderate	44	64	2.2%	3.0%	48	24	S2	17.35	19.27	0.25%	TRUE	19.32	49.24
High School	Moderate	45	65	2.2%	3.0%	48	24	S2	17.35	19.32	0.25%	TRUE	19.36	50.45
High School	Strong	1	21	2.2%	3.0%	48	30	S2	17.35	-	0.25%	TRUE	17.35	17.35
High School	Strong	2	22	2.2%	3.0%	48	30	S2	17.35	17.35	0.25%	TRUE	17.39	17.78
High School	Strong	3	23	2.2%	3.0%	48	30	S2	17.35	17.39	0.25%	TRUE	17.44	18.21
High School	Strong	4	24	2.2%	3.0%	48	30	S2	17.35	17.44	0.25%	TRUE	17.48	18.66
High School	Strong	5	25	2.2%	3.0%	48	30	S2	17.35	17.48	0.25%	TRUE	17.52	19.12
High School	Strong	6	26	2.2%	3.0%	48	35	32,300	32,300.00	17.52	0.25%	FALSE	32,300.00	36,012.81
High School	Strong	7	27	2.2%	3.0%	48	35	32,300	32,300.00	32,300.00	0.25%	TRUE	32,380.75	36,897.10
High School	Strong	8	28	2.2%	3.0%	48	35	32,300	32,300.00	32,380.75	0.25%	TRUE	32,461.70	37,803.11
High School	Strong	9	29	2.2%	3.0%	48	35	32,300	32,300.00	32,461.70	0.25%	TRUE	32,542.86	38,731.37
High School	Strong	10	30	2.2%	3.0%	48	35	32,300	32,300.00	32,542.86	0.25%	TRUE	32,624.21	39,682.42
High School	Strong	11	31	2.2%	3.0%	48	35	32,300	32,300.00	32,624.21	0.25%	TRUE	32,705.77	40,656.82
High School	Strong	12	32	2.2%	3.0%	48	35	32,300	32,300.00	32,705.77	0.25%	TRUE	32,787.54	41,655.15
High School	Strong	13	33	2.2%	3.0%	48	35	32,300	32,300.00	32,787.54	0.25%	TRUE	32,869.51	42,677.99
High School	Strong	14	34	2.2%	3.0%	48	35	32,300	32,300.00	32,869.51	0.25%	TRUE	32,951.68	43,725.95
High School	Strong	15	35	2.2%	3.0%	48	35	32,300	32,300.00	32,951.68	0.25%	TRUE	33,034.06	44,799.64
High School	Strong	16	36	2.2%	3.0%	48	35	32,300	32,300.00	33,034.06	0.25%	TRUE	33,116.65	45,899.69
High School	Strong	17	37	2.2%	3.0%	48	35	32,300	32,300.00	33,116.65	0.25%	TRUE	33,199.44	47,026.76
High School	Strong	18	38	2.2%	3.0%	48	35	32,300	32,300.00	33,199.44	0.25%	TRUE	33,282.44	48,181.50
High School	Strong	19	39	2.2%	3.0%	48	35	32,300	32,300.00	33,282.44	0.25%	TRUE	33,365.64	49,364.60
High School	Strong	20	40	2.2%	3.0%	48	35	32,300	32,300.00	33,365.64	0.25%	TRUE	33,449.06	50,576.75
High School	Strong	21	41	2.2%	3.0%	48	35	32,300	32,300.00	33,449.06	0.25%	TRUE	33,532.68	51,818.66
High School	Strong	22	42	2.2%	3.0%	48	35	32,300	32,300.00	33,532.68	0.25%	TRUE	33,616.51	53,091.06
High School	Strong	23	43	2.2%	3.0%	48	35	32,300	32,300.00	33,616.51	0.25%	TRUE	33,700.55	54,394.72
High School	Strong	24	44	2.2%	3.0%	48	35	32,300	32,300.00	33,700.55	0.25%	TRUE	33,784.80	55,730.38
High School	Strong	25	45	2.2%	3.0%	48	35	32,300	32,300.00	33,784.80	0.25%	TRUE	33,869.26	57,098.84
High School	Strong	26	46	2.2%	3.0%	48	35	32,300	32,300.00	33,869.26	0.25%	TRUE	33,953.94	58,500.90
High School	Strong	27	47	2.2%	3.0%	48	35	32,300	32,300.00	33,953.94	0.25%	TRUE	34,038.82	59,937.39
High School	Strong	28	48	2.2%	3.0%	48	35	32,300	32,300.00	34,038.82	0.25%	TRUE	34,123.92	61,409.15
High School	Strong	29	49	2.2%	3.0%	48	35	32,300	32,300.00	34,123.92	0.25%	TRUE	34,209.23	62,917.05
High School	Strong	30	50	2.2%	3.0%	48	35	32,300	32,300.00	34,209.23	0.25%	TRUE	34,294.75	64,461.98
High School	Strong	31	51	2.2%	3.0%	48	35	32,300	32,300.00	34,294.75	0.25%	TRUE	34,380.49	66,044.85
High School	Strong	32	52	2.2%	3.0%	48	35	32,300	32,300.00	34,380.49	0.25%	TRUE	34,466.44	67,666.58
High School	Strong	33	53	2.2%	3.0%	48	35	32,300	32,300.00	34,466.44	0.25%	TRUE	34,552.61	69,328.13
High School	Strong	34	54	2.2%	3.0%	48	35	32,300	32,300.00	34,552.61	0.25%	TRUE	34,638.99	71,030.48
High School	Strong	35	55	2.2%	3.0%	48	35	32,300	32,300.00	34,638.99	0.25%	TRUE	34,725.59	72,774.64
High School	Strong	36	56	2.2%	3.0%	48	35	32,300	32,300.00	34,725.59	0.25%	TRUE	34,812.40	74,561.62
High School	Strong	37	57	2.2%	3.0%	48	35	32,300	32,300.00	34,812.40	0.25%	TRUE	34,899.43	76,392.48
High School	Strong	38	58	2.2%	3.0%	48	35	32,300	32,300.00	34,899.43	0.25%	TRUE	34,986.68	78,268.29
High School	Strong	39	59	2.2%	3.0%	48	35	32,300	32,300.00	34,986.68	0.25%	TRUE	35,074.15	80,190.17
High School	Strong	40	60	2.2%	3.0%	48	35	32,300	32,300.00	35,074.15	0.25%	TRUE	35,161.83	82,159.24
High School	Strong	41	61	2.2%	3.0%	48	35	32,300	32,300.00	35,161.83	0.25%	TRUE	35,249.74	84,176.66
High School	Strong	42	62	2.2%	3.0%	48	35	32,300	32,300.00	35,249.74	0.25%	TRUE	35,337.86	86,243.62
High School	Strong	43	63	2.2%	3.0%	48	35	32,300	32,300.00	35,337.86	0.25%	TRUE	35,426.20	88,361.33
High School	Strong	44	64	2.2%	3.0%	48	35	32,300	32,300.00	35,426.20	0.25%	TRUE	35,514.77	90,531.04
High School	Strong	45	65	2.2%	3.0%	48	35	32,300	32,300.00	35,514.77	0.25%	TRUE	35,603.56	92,754.03
University	Baseline	1	17	2.2%	3.0%			NONE	-	-	0.25%	TRUE	-	-
University	Baseline	2	18	2.2%	3.0%			NONE	-	-	0.25%	TRUE	-	-
University	Baseline	3	19	2.2%	3.0%			NONE	-	-	0.25%	TRUE	-	-
University	Baseline	4	20	2.2%	3.0%			NONE	-	-	0.25%	TRUE	-	-
University	Baseline	5	21	2.2%	3.0%			NONE	-	-	0.25%	TRUE	-	-
University	Baseline	6	22	2.2%	3.0%	22	25	MW	15.50	-	0.25%	FALSE	15.50	17.28
University	Baseline	7	23	2.2%	3.0%	48	25	MW	15.50	15.50	0.25%	TRUE	15.54	17.71
University	Baseline	8	24	2.2%	3.0%	48	25	S2	17.35	15.54	0.25%	TRUE	15.58	18.14
University	Baseline	9	25	2.2%	3.0%	48	35	S2	17.35	15.58	0.25%	TRUE	15.62	18.59
University	Baseline	10	26	2.2%	3.0%	52	35	45,000	45,000.00	15.62	0.25%	FALSE	45,000.00	54,735.69
University	Baseline	11	27	2.2%	3.0%	52	35	45,000	45,000.00	45,000.00	0.25%	TRUE	45,112.50	56,079.72

EARNINGS VARIABLES VALUES

Persona	Scenario	Year	Age	Inflation	Discount	Wks/Yr	Hrs /Wk	Wage Type	Nominal Wage or Salary	Last Year Nominal Wage or Salary	Wage Progression	Continue from Previous Year	Progressed Wage	Progressed Wage with Inflation
University	Baseline	12	28	2.2%	3.0%	52	35	45,000	45,000.00	45,112.50	0.25%	TRUE	45,225.28	57,456.76
University	Baseline	13	29	2.2%	3.0%	52	35	45,000	45,000.00	45,225.28	0.25%	TRUE	45,338.34	58,867.61
University	Baseline	14	30	2.2%	3.0%	52	35	45,000	45,000.00	45,338.34	0.25%	TRUE	45,451.69	60,313.10
University	Baseline	15	31	2.2%	3.0%	52	35	45,000	45,000.00	45,451.69	0.25%	TRUE	45,565.32	61,794.09
University	Baseline	16	32	2.2%	3.0%	52	35	45,000	45,000.00	45,565.32	0.25%	TRUE	45,679.23	63,311.45
University	Baseline	17	33	2.2%	3.0%	52	35	45,000	45,000.00	45,679.23	0.25%	TRUE	45,793.43	64,866.06
University	Baseline	18	34	2.2%	3.0%	52	35	45,000	45,000.00	45,793.43	0.25%	TRUE	45,907.91	66,458.85
University	Baseline	19	35	2.2%	3.0%	52	35	45,000	45,000.00	45,907.91	0.25%	TRUE	46,022.68	68,090.74
University	Baseline	20	36	2.2%	3.0%	52	35	45,000	45,000.00	46,022.68	0.25%	TRUE	46,137.74	69,762.71
University	Baseline	21	37	2.2%	3.0%	52	35	45,000	45,000.00	46,137.74	0.25%	TRUE	46,253.09	71,475.73
University	Baseline	22	38	2.2%	3.0%	52	35	45,000	45,000.00	46,253.09	0.25%	TRUE	46,368.72	73,230.82
University	Baseline	23	39	2.2%	3.0%	52	35	45,000	45,000.00	46,368.72	0.25%	TRUE	46,484.64	75,029.00
University	Baseline	24	40	2.2%	3.0%	52	35	45,000	45,000.00	46,484.64	0.25%	TRUE	46,600.85	76,871.34
University	Baseline	25	41	2.2%	3.0%	52	35	45,000	45,000.00	46,600.85	0.25%	TRUE	46,717.35	78,758.92
University	Baseline	26	42	2.2%	3.0%	52	35	45,000	45,000.00	46,717.35	0.25%	TRUE	46,834.15	80,692.84
University	Baseline	27	43	2.2%	3.0%	52	35	45,000	45,000.00	46,834.15	0.25%	TRUE	46,951.23	82,674.25
University	Baseline	28	44	2.2%	3.0%	52	35	45,000	45,000.00	46,951.23	0.25%	TRUE	47,068.61	84,704.32
University	Baseline	29	45	2.2%	3.0%	52	35	45,000	45,000.00	47,068.61	0.25%	TRUE	47,186.28	86,784.24
University	Baseline	30	46	2.2%	3.0%	52	35	45,000	45,000.00	47,186.28	0.25%	TRUE	47,304.25	88,915.22
University	Baseline	31	47	2.2%	3.0%	52	35	45,000	45,000.00	47,304.25	0.25%	TRUE	47,422.51	91,098.54
University	Baseline	32	48	2.2%	3.0%	52	35	45,000	45,000.00	47,422.51	0.25%	TRUE	47,541.06	93,335.46
University	Baseline	33	49	2.2%	3.0%	52	35	45,000	45,000.00	47,541.06	0.25%	TRUE	47,659.92	95,627.31
University	Baseline	34	50	2.2%	3.0%	52	35	45,000	45,000.00	47,659.92	0.25%	TRUE	47,779.07	97,975.44
University	Baseline	35	51	2.2%	3.0%	52	35	45,000	45,000.00	47,779.07	0.25%	TRUE	47,898.51	100,381.23
University	Baseline	36	52	2.2%	3.0%	52	35	45,000	45,000.00	47,898.51	0.25%	TRUE	48,018.26	102,846.09
University	Baseline	37	53	2.2%	3.0%	52	35	45,000	45,000.00	48,018.26	0.25%	TRUE	48,138.31	105,371.47
University	Baseline	38	54	2.2%	3.0%	52	35	45,000	45,000.00	48,138.31	0.25%	TRUE	48,258.65	107,958.87
University	Baseline	39	55	2.2%	3.0%	52	35	45,000	45,000.00	48,258.65	0.25%	TRUE	48,379.30	110,609.80
University	Baseline	40	56	2.2%	3.0%	52	35	45,000	45,000.00	48,379.30	0.25%	TRUE	48,500.25	113,325.82
University	Baseline	41	57	2.2%	3.0%	52	35	45,000	45,000.00	48,500.25	0.25%	TRUE	48,621.50	116,108.54
University	Baseline	42	58	2.2%	3.0%	52	35	45,000	45,000.00	48,621.50	0.25%	TRUE	48,743.05	118,959.59
University	Baseline	43	59	2.2%	3.0%	52	35	45,000	45,000.00	48,743.05	0.25%	TRUE	48,864.91	121,880.64
University	Baseline	44	60	2.2%	3.0%	52	35	45,000	45,000.00	48,864.91	0.25%	TRUE	48,987.07	124,873.42
University	Baseline	45	61	2.2%	3.0%	52	35	45,000	45,000.00	48,987.07	0.25%	TRUE	49,109.54	127,939.68
University	Baseline	46	62	2.2%	3.0%	52	35	45,000	45,000.00	49,109.54	0.25%	TRUE	49,232.31	131,081.24
University	Baseline	47	63	2.2%	3.0%	52	35	45,000	45,000.00	49,232.31	0.25%	TRUE	49,355.39	134,299.94
University	Baseline	48	64	2.2%	3.0%	52	35	45,000	45,000.00	49,355.39	0.25%	TRUE	49,478.78	137,597.68
University	Baseline	49	65	2.2%	3.0%	52	35	45,000	45,000.00	49,478.78	0.25%	TRUE	49,602.48	140,976.39
University	Moderate	1	17	2.2%	3.0%	8	35	MW	15.50	-	0.25%	TRUE	15.50	15.50
University	Moderate	2	18	2.2%	3.0%	8	35	MW	15.50	15.50	0.25%	TRUE	15.54	15.88
University	Moderate	3	19	2.2%	3.0%	8	35	MW	15.50	15.54	0.25%	TRUE	15.58	16.27
University	Moderate	4	20	2.2%	3.0%	8	35	MW	15.50	15.58	0.25%	TRUE	15.62	16.67
University	Moderate	5	21	2.2%	3.0%	8	35	MW	15.50	15.62	0.25%	TRUE	15.66	17.08
University	Moderate	6	22	2.2%	3.0%	52	35	55,000	55,000.00	15.66	0.25%	FALSE	55,000.00	61,322.12
University	Moderate	7	23	2.2%	3.0%	52	35	55,000	55,000.00	55,000.00	0.25%	TRUE	55,137.50	62,827.89
University	Moderate	8	24	2.2%	3.0%	52	35	55,000	55,000.00	55,137.50	0.25%	TRUE	55,275.34	64,370.62
University	Moderate	9	25	2.2%	3.0%	52	35	55,000	55,000.00	55,275.34	0.25%	TRUE	55,413.53	65,951.25
University	Moderate	10	26	2.2%	3.0%	52	35	55,000	55,000.00	55,413.53	0.25%	TRUE	55,552.07	67,570.68
University	Moderate	11	27	2.2%	3.0%	52	35	55,000	55,000.00	55,552.07	0.25%	TRUE	55,690.95	69,229.88
University	Moderate	12	28	2.2%	3.0%	52	35	55,000	55,000.00	55,690.95	0.25%	TRUE	55,830.17	70,929.82
University	Moderate	13	29	2.2%	3.0%	52	35	55,000	55,000.00	55,830.17	0.25%	TRUE	55,969.75	72,671.50
University	Moderate	14	30	2.2%	3.0%	52	35	55,000	55,000.00	55,969.75	0.25%	TRUE	56,109.67	74,455.95
University	Moderate	15	31	2.2%	3.0%	52	35	55,000	55,000.00	56,109.67	0.25%	TRUE	56,249.95	76,284.21
University	Moderate	16	32	2.2%	3.0%	52	35	55,000	55,000.00	56,249.95	0.25%	TRUE	56,390.57	78,157.37
University	Moderate	17	33	2.2%	3.0%	52	35	55,000	55,000.00	56,390.57	0.25%	TRUE	56,531.55	80,076.52
University	Moderate	18	34	2.2%	3.0%	52	35	55,000	55,000.00	56,531.55	0.25%	TRUE	56,672.88	82,042.80
University	Moderate	19	35	2.2%	3.0%	52	35	55,000	55,000.00	56,672.88	0.25%	TRUE	56,814.56	84,057.36
University	Moderate	20	36	2.2%	3.0%	52	35	55,000	55,000.00	56,814.56	0.25%	TRUE	56,956.60	86,121.39
University	Moderate	21	37	2.2%	3.0%	52	35	55,000	55,000.00	56,956.60	0.25%	TRUE	57,098.99	88,236.10
University	Moderate	22	38	2.2%	3.0%	52	35	55,000	55,000.00	57,098.99	0.25%	TRUE	57,241.74	90,402.74
University	Moderate	23	39	2.2%	3.0%	52	35	55,000	55,000.00	57,241.74	0.25%	TRUE	57,384.84	92,622.58
University	Moderate	24	40	2.2%	3.0%	52	35	55,000	55,000.00	57,384.84	0.25%	TRUE	57,528.30	94,896.93
University	Moderate	25	41	2.2%	3.0%	52	35	55,000	55,000.00	57,528.30	0.25%	TRUE	57,672.12	97,227.12
University	Moderate	26	42	2.2%	3.0%	52	35	55,000	55,000.00	57,672.12	0.25%	TRUE	57,816.30	99,614.53
University	Moderate	27	43	2.2%	3.0%	52	35	55,000	55,000.00	57,816.30	0.25%	TRUE	57,960.84	102,060.57
University	Moderate	28	44	2.2%	3.0%	52	35	55,000	55,000.00	57,960.84	0.25%	TRUE	58,105.75	104,566.67
University	Moderate	29	45	2.2%	3.0%	52	35	55,000	55,000.00	58,105.75	0.25%	TRUE	58,251.01	107,134.30
University	Moderate	30	46	2.2%	3.0%	52	35	55,000	55,000.00	58,251.01	0.25%	TRUE	58,396.64	109,764.98
University	Moderate	31	47	2.2%	3.0%	52	35	55,000	55,000.00	58,396.64	0.25%	TRUE	58,542.63	112,460.26
University	Moderate	32	48	2.2%	3.0%	52	35	55,000	55,000.00	58,542.63	0.25%	TRUE	58,688.99	115,221.73
University	Moderate	33	49	2.2%	3.0%	52	35	55,000	55,000.00	58,688.99	0.25%	TRUE	58,835.71	118,050.99
University	Moderate	34	50	2.2%	3.0%	52	35	55,000	55,000.00	58,835.71	0.25%	TRUE	58,982.80	120,949.74
University	Moderate	35	51	2.2%	3.0%	52	35	55,000	55,000.00	58,982.80	0.25%	TRUE	59,130.25	123,919.66

EARNINGS VARIABLES VALUES

Persona	Scenario	Year	Age	Inflation	Discount	Wks/Yr	Hrs /Wk	Wage Type	Nominal Wage or Salary	Last Year Nominal Wage or Salary	Wage Progression	Continue from Previous Year	Progressed Wage	Progressed Wage with Inflation
University	Moderate	36	52	2.2%	3.0%	52	35	55,000	55,000.00	59,130.25	0.25%	TRUE	59,278.08	126,962.50
University	Moderate	37	53	2.2%	3.0%	52	35	55,000	55,000.00	59,278.08	0.25%	TRUE	59,426.28	130,080.07
University	Moderate	38	54	2.2%	3.0%	52	35	55,000	55,000.00	59,426.28	0.25%	TRUE	59,574.84	133,274.18
University	Moderate	39	55	2.2%	3.0%	52	35	55,000	55,000.00	59,574.84	0.25%	TRUE	59,723.78	136,546.73
University	Moderate	40	56	2.2%	3.0%	52	35	55,000	55,000.00	59,723.78	0.25%	TRUE	59,873.09	139,899.64
University	Moderate	41	57	2.2%	3.0%	52	35	55,000	55,000.00	59,873.09	0.25%	TRUE	60,022.77	143,334.87
University	Moderate	42	58	2.2%	3.0%	52	35	55,000	55,000.00	60,022.77	0.25%	TRUE	60,172.83	146,854.46
University	Moderate	43	59	2.2%	3.0%	52	35	55,000	55,000.00	60,172.83	0.25%	TRUE	60,323.26	150,460.47
University	Moderate	44	60	2.2%	3.0%	52	35	55,000	55,000.00	60,323.26	0.25%	TRUE	60,474.07	154,155.03
University	Moderate	45	61	2.2%	3.0%	52	35	55,000	55,000.00	60,474.07	0.25%	TRUE	60,625.25	157,940.31
University	Moderate	46	62	2.2%	3.0%	52	35	55,000	55,000.00	60,625.25	0.25%	TRUE	60,776.82	161,818.53
University	Moderate	47	63	2.2%	3.0%	52	35	55,000	55,000.00	60,776.82	0.25%	TRUE	60,928.76	165,791.98
University	Moderate	48	64	2.2%	3.0%	52	35	55,000	55,000.00	60,928.76	0.25%	TRUE	61,081.08	169,863.01
University	Moderate	49	65	2.2%	3.0%	52	35	55,000	55,000.00	61,081.08	0.25%	TRUE	61,233.78	174,033.99
University	Strong	1	17	2.2%	3.0%	16	35	S3	23.00	-	0.25%	TRUE	23.00	23.00
University	Strong	2	18	2.2%	3.0%	16	35	S3	23.00	23.00	0.25%	TRUE	23.06	23.56
University	Strong	3	19	2.2%	3.0%	16	35	S3	23.00	23.06	0.25%	TRUE	23.12	24.14
University	Strong	4	20	2.2%	3.0%	16	35	S3	23.00	23.12	0.25%	TRUE	23.17	24.74
University	Strong	5	21	2.2%	3.0%	16	35	S3	23.00	23.17	0.25%	TRUE	23.23	25.34
University	Strong	6	22	2.2%	3.0%	52	35	65,000	65,000.00	23.23	0.25%	FALSE	65,000.00	72,471.60
University	Strong	7	23	2.2%	3.0%	52	35	65,000	65,000.00	65,000.00	0.25%	TRUE	65,162.50	74,251.14
University	Strong	8	24	2.2%	3.0%	52	35	65,000	65,000.00	65,162.50	0.25%	TRUE	65,325.41	76,074.37
University	Strong	9	25	2.2%	3.0%	52	35	65,000	65,000.00	65,325.41	0.25%	TRUE	65,488.72	77,942.38
University	Strong	10	26	2.2%	3.0%	52	35	65,000	65,000.00	65,488.72	0.25%	TRUE	65,652.44	79,856.26
University	Strong	11	27	2.2%	3.0%	52	35	65,000	65,000.00	65,652.44	0.25%	TRUE	65,816.57	81,817.13
University	Strong	12	28	2.2%	3.0%	52	35	65,000	65,000.00	65,816.57	0.25%	TRUE	65,981.11	83,826.15
University	Strong	13	29	2.2%	3.0%	52	35	65,000	65,000.00	65,981.11	0.25%	TRUE	66,146.07	85,884.50
University	Strong	14	30	2.2%	3.0%	52	35	65,000	65,000.00	66,146.07	0.25%	TRUE	66,311.43	87,993.39
University	Strong	15	31	2.2%	3.0%	52	35	65,000	65,000.00	66,311.43	0.25%	TRUE	66,477.21	90,154.07
University	Strong	16	32	2.2%	3.0%	52	35	65,000	65,000.00	66,477.21	0.25%	TRUE	66,643.40	92,367.80
University	Strong	17	33	2.2%	3.0%	52	35	65,000	65,000.00	66,643.40	0.25%	TRUE	66,810.01	94,635.89
University	Strong	18	34	2.2%	3.0%	52	35	65,000	65,000.00	66,810.01	0.25%	TRUE	66,977.04	96,959.68
University	Strong	19	35	2.2%	3.0%	52	35	65,000	65,000.00	66,977.04	0.25%	TRUE	67,144.48	99,340.52
University	Strong	20	36	2.2%	3.0%	52	35	65,000	65,000.00	67,144.48	0.25%	TRUE	67,312.34	101,779.83
University	Strong	21	37	2.2%	3.0%	52	35	65,000	65,000.00	67,312.34	0.25%	TRUE	67,480.62	104,279.03
University	Strong	22	38	2.2%	3.0%	52	35	65,000	65,000.00	67,480.62	0.25%	TRUE	67,649.32	106,839.60
University	Strong	23	39	2.2%	3.0%	52	35	65,000	65,000.00	67,649.32	0.25%	TRUE	67,818.45	109,463.05
University	Strong	24	40	2.2%	3.0%	52	35	65,000	65,000.00	67,818.45	0.25%	TRUE	67,987.99	112,150.92
University	Strong	25	41	2.2%	3.0%	52	35	65,000	65,000.00	67,987.99	0.25%	TRUE	68,157.96	114,904.78
University	Strong	26	42	2.2%	3.0%	52	35	65,000	65,000.00	68,157.96	0.25%	TRUE	68,328.36	117,726.27
University	Strong	27	43	2.2%	3.0%	52	35	65,000	65,000.00	68,328.36	0.25%	TRUE	68,499.18	120,617.04
University	Strong	28	44	2.2%	3.0%	52	35	65,000	65,000.00	68,499.18	0.25%	TRUE	68,670.43	123,578.79
University	Strong	29	45	2.2%	3.0%	52	35	65,000	65,000.00	68,670.43	0.25%	TRUE	68,842.10	126,613.27
University	Strong	30	46	2.2%	3.0%	52	35	65,000	65,000.00	68,842.10	0.25%	TRUE	69,014.21	129,722.25
University	Strong	31	47	2.2%	3.0%	52	35	65,000	65,000.00	69,014.21	0.25%	TRUE	69,186.74	132,907.58
University	Strong	32	48	2.2%	3.0%	52	35	65,000	65,000.00	69,186.74	0.25%	TRUE	69,359.71	136,171.13
University	Strong	33	49	2.2%	3.0%	52	35	65,000	65,000.00	69,359.71	0.25%	TRUE	69,533.11	139,514.81
University	Strong	34	50	2.2%	3.0%	52	35	65,000	65,000.00	69,533.11	0.25%	TRUE	69,706.94	142,940.60
University	Strong	35	51	2.2%	3.0%	52	35	65,000	65,000.00	69,706.94	0.25%	TRUE	69,881.21	146,450.50
University	Strong	36	52	2.2%	3.0%	52	35	65,000	65,000.00	69,881.21	0.25%	TRUE	70,055.91	150,046.60
University	Strong	37	53	2.2%	3.0%	52	35	65,000	65,000.00	70,055.91	0.25%	TRUE	70,231.05	153,730.99
University	Strong	38	54	2.2%	3.0%	52	35	65,000	65,000.00	70,231.05	0.25%	TRUE	70,406.63	157,505.85
University	Strong	39	55	2.2%	3.0%	52	35	65,000	65,000.00	70,406.63	0.25%	TRUE	70,582.65	161,373.41
University	Strong	40	56	2.2%	3.0%	52	35	65,000	65,000.00	70,582.65	0.25%	TRUE	70,759.10	165,335.94
University	Strong	41	57	2.2%	3.0%	52	35	65,000	65,000.00	70,759.10	0.25%	TRUE	70,936.00	169,395.76
University	Strong	42	58	2.2%	3.0%	52	35	65,000	65,000.00	70,936.00	0.25%	TRUE	71,113.34	173,555.27
University	Strong	43	59	2.2%	3.0%	52	35	65,000	65,000.00	71,113.34	0.25%	TRUE	71,291.12	177,816.92
University	Strong	44	60	2.2%	3.0%	52	35	65,000	65,000.00	71,291.12	0.25%	TRUE	71,469.35	182,183.22
University	Strong	45	61	2.2%	3.0%	52	35	65,000	65,000.00	71,469.35	0.25%	TRUE	71,648.03	186,656.73
University	Strong	46	62	2.2%	3.0%	52	35	65,000	65,000.00	71,648.03	0.25%	TRUE	71,827.15	191,240.08
University	Strong	47	63	2.2%	3.0%	52	35	65,000	65,000.00	71,827.15	0.25%	TRUE	72,006.71	195,935.98
University	Strong	48	64	2.2%	3.0%	52	35	65,000	65,000.00	72,006.71	0.25%	TRUE	72,186.73	200,747.19
University	Strong	49	65	2.2%	3.0%	52	35	65,000	65,000.00	72,186.73	0.25%	TRUE	72,367.20	205,676.54
