# Supplementary material for: Funding employment inclusion for Ontario youth with disabilities: a theoretical cost-benefit model
Source: Front Sociol. 2024 Apr 10;9:1281088. doi: 10.3389/fsoc.2024.1281088 (PMC11041630; doi:10.3389/fsoc.2024.1281088)
Supplement: Supplementary Appendix C — Full description. [file Data_Sheet_3.PDF]

Funding Employment Inclusion for Ontario Youth with Disabilities: A Cost-Benefit Model

Bowman, McDougall, Doucet, Pooran, Xu & Campbell

Supplementary Appendix C. Full description of sensitivity analyses

Four sensitivity analyses were run, varying alternately the inflation rate, discount rate, inclusion of RRSP contributions (university persona only), and exclusion of the DTC. We note that any of the variables in the model can be altered to see the effect on the return, down to any of the five earnings variables for a specific year for a given persona-scenario combination. The four sensitivity analyses were chosen as most relevant to the topic and current economic climate. The four sensitivity analysis scenarios are presented in Table C1, and illustrate that there remains an economic gain to government under a variety of conditions.

Table C1. Summary of sensitivity analyses

		Baseline			Moderate			Strong		
		Cash Flow	Return $	Return %	Cash Flow	Return $	Return %	Cash Flow	Return $	Return %
High School	Main	-329,082	N/A	N/A	-203,719	125,363	684%	-33,490	295,592	1,747%
	↑ Inflation	-350,996	N/A	N/A	-220,522	130,474	715%	-8,666	342,330	2,040%
	↑ Discount Rate	-176,430	N/A	N/A	-124,200	52,230	226%	-42,420	134,010	738%
	RRSP	N/A	N/A	N/A	N/A	N/A	N/A	N/A	N/A	N/A
	No DTC	-301,873	N/A	N/A	-149,963	151,910	849%	48,605	350,478	2,090%
University	Main	44,613	N/A	N/A	184,252	139,639	734%	286,043	241,431	1,341%
	↑ Inflation	146,148	N/A	N/A	356,367	210,220	1,155%	526,578	380,430	2,171%
	↑ Discount Rate	-37,201	N/A	N/A	19,958	57,159	249%	50,319	87,520	435%
	RRSP	-14,142	N/A	N/A	77,998	92,139	450%	160,492	174,634	942%
	No DTC	105,211	N/A	N/A	250,485	145,274	767%	352,276	247,065	1,375%

Sensitivity Analysis 1: Inflation Rate

We first explored the impact of inflation on the outcomes of our model, due to the current economic relevance of rising inflation rates. In this example, the inflation rate was adjusted to 4.0%, chosen as a conservative analysis point that sits slightly higher than the historical average of 3.8% (World Data, n.d.). All other variables were maintained as defined above. For the high school persona, a higher inflation rate still resulted in a return of $130,474 (715%) in the moderate outcome scenario, and $342,330 (2,040%) in the strong outcome scenario over the baseline scenario. For the university persona, it resulted in a return of $210,220 (1,155%) in the moderate outcome scenario, and $380,430 (2,171%) in the strong outcome scenario over baseline. This sensitivity analysis demonstrated that the model is sensitive to changes in inflation rates, and demonstrates that the return to government increases as inflation increases.

Sensitivity Analysis 2: Discount Rate

We next varied the discount rate from 3.0% in the main model application (as is usually applied to social programs) to a rate of 8.0%, representing the typical private rate of investment (Treasury Board of Canada, 2007). For the high school persona, an 8% discount rate resulted in a return of $52,230 (226%) in the moderate outcome scenario, and $134,010 (738%) in the strong outcome scenario over baseline. For the university persona, it resulted in a return of $57,159 (249%) in the moderate outcome scenario, and $87,520 (435%) in the strong outcome scenario over baseline. The analysis demonstrates that the higher the discount rate, the lower the return to government, for the simple reason that the positive return in the moderate and strong outcome scenarios (as compared to the baseline) grow faster in the later years, which, by definition, are more impacted by higher discount rates when converting to present-day dollars.

Sensitivity Analysis 3: Registered Retirement Savings Plan (RRSP)

In the model above, we assumed that our personas would not contribute to a registered, tax-exempt retirement investment plan (RRSP) annually. To explore the potential impact of contribution to such a savings plan, we applied a maximum annual RRSP contribution to the university persona, calculated based on 18% of annual income. This was only applied to the university persona due to the likelihood of

relevance of contribution for the higher earning group. RRSP contributions will reduce the return to government within the model's scope, however, some or all of the RRSP contributions may be taxed when the amounts are withdrawn in retirement, which would then increase the return to government. Regardless, the return is still positive for government even within the model's scope and assuming *maximum* annual RRSP contributions, which, for the University persona, resulted in a return of $92,139 (450%) in the moderate outcome scenario, and $174,634 (942%) in the strong outcome scenario over baseline.

Sensitivity Analysis 4: Disability Tax Credit

Finally, we assessed the costs and benefits of *not* claiming the DTC to explore the model's sensitivity to the application of tax credits, keeping all other variables as defined above. This was specifically undertaken to reflect the fact that many individuals in Ontario do not claim the tax credit in real life due to the burden of the application process. For the high school persona, not claiming the DTC resulted in a return of $151,910 (849%) in the moderate outcome scenario, and $350,478 (2,090%) in the strong outcome scenario over baseline. For the university persona, it resulted in a return of $145,274 (767%) in the moderate outcome scenario, and $247,065 (1,375%) in the strong outcome scenario over baseline. Our model demonstrated increased net societal-level benefits when no DTC was claimed on the tax return.

References

Treasury Board of Canada. (2007). Canadian Cost-Benefit Analysis Guide: Regulatory Proposals *In:* SECRETARIAT, T. B. O. C. (ed.). Canada: Her Majesty the Queen in Right of Canada.

World Data. (n.d.). *Inflation Rates in Canada* [Online]. WorldDada.info. Available: https://www.worlddata.info/america/canada/inflation-rates.php [Accessed].
